# Relationship of Signaling Pathways between RKIP Expression and the Inhibition of EMT-Inducing Transcription Factors SNAIL1/2, TWIST1/2 and ZEB1/2

**DOI:** 10.3390/cancers16183180

**Published:** 2024-09-17

**Authors:** Andrew Bustamante, Stavroula Baritaki, Apostolos Zaravinos, Benjamin Bonavida

**Affiliations:** 1 Department of Microbiology, Immunology & Molecular Genetics, David Geffen School of Medicine, Jonsson Comprehensive Cancer Center, University of California at Los Angeles, Los Angeles, CA 90095, USA; bustamante23@g.ucla.edu; 2 Laboratory of Experimental Oncology, Division of Surgery, School of Medicine, University of Crete, 71003 Heraklion, Greece; baritaks@uoc.gr; 3 Cancer Genetics, Genomics and Systems Biology Laboratory, Basic and Translational Cancer Research Center (BTCRC), Nicosia 1516, Cyprus; a.zaravinos@euc.ac.cy; 4 Department of Life Sciences, School of Sciences, European University Cyprus, Nicosia 1516, Cyprus

**Keywords:** cancer, EMT, RKIP, cross-talk pathways, SNAIL, TWIST, ZEB, resistance, targeting

## Abstract

**Simple Summary:**

Cancer is a deadly disease if it is not treated early with various conventional therapies that include surgery, radiation, chemotherapy and immunotherapy. These treatments manage a large percentage of patients with various cancer types. Normally, we do not develop cancer due to an inherent immune mechanism that is able to suppress its development. However, cancer can also develop means to counter the suppression mechanisms to grow and survive. While the suppressive mechanism is normally dominant in most humans, it also fails in many instances, allowing the cancer to grow and resist treatments. Accordingly, if one is able to use agents that can augment the suppressive mechanisms in cancer, it may result in the elimination of the tumor. In this study, we have addressed factors that support tumor growth and suggested approaches to target these factors that can lead to the destruction of the tumor.

**Abstract:**

Untreated primary carcinomas often lead to progression, invasion and metastasis, a process that involves the epithelial-to-mesenchymal transition (EMT). Several transcription factors (TFs) mediate the development of EMT, including SNAIL1/SNAIL2, TWIST1/TWIST2 and ZEB1/ZEB2, which are overexpressed in various carcinomas along with the under expression of the metastasis suppressor Raf Kinase Inhibitor Protein (RKIP). Overexpression of RKIP inhibits EMT and the above associated TFs. We, therefore, hypothesized that there are inhibitory cross-talk signaling pathways between RKIP and these TFs. Accordingly, we analyzed the various properties and biomarkers associated with the epithelial and mesenchymal tissues and the various molecular signaling pathways that trigger the EMT phenotype such as the TGF-β, the RTK and the Wnt pathways. We also presented the various functions and the transcriptional, post-transcriptional and epigenetic regulations for the expression of each of the EMT TFs. Likewise, we describe the transcriptional, post-transcriptional and epigenetic regulations of RKIP expression. Various signaling pathways mediated by RKIP, including the Raf/MEK/ERK pathway, inhibit the TFs associated with EMT and the stabilization of epithelial E-Cadherin expression. The inverse relationship between RKIP and the TF expressions and the cross-talks were further analyzed by bioinformatic analysis. High mRNA levels of RKIP correlated negatively with those of SNAIL1, SNAIL2, TWIST1, TWIST2, ZEB1, and ZEB2 in several but not all carcinomas. However, in these carcinomas, high levels of RKIP were associated with good prognosis, whereas high levels of the above transcription factors were associated with poor prognosis. Based on the inverse relationship between RKIP and EMT TFs, it is postulated that the expression level of RKIP in various carcinomas is clinically relevant as both a prognostic and diagnostic biomarker. In addition, targeting RKIP induction by agonists, gene therapy and immunotherapy will result not only in the inhibition of EMT and metastases in carcinomas, but also in the inhibition of tumor growth and reversal of resistance to various therapeutic strategies. However, such targeting strategies must be better investigated as a result of tumor heterogeneities and inherent resistance and should be better adapted as personalized medicine.

## 1. Introduction

The epithelial-to-mesenchymal transition (EMT) is a biologically critical process that enables epithelial cells to abandon their traditional epithelial function and adopt mesenchymal features. EMT functions as a spectrum for epithelial cells, enabling them to either partially or fully convert into mesenchymal cells and to revert back [1]. The process of reverting back to the epithelial phenotype is known as the mesenchymal–epithelial transition (MET). Both EMT and MET are fundamental processes in embryonic development and tissue repair, while they also confer malignant cell properties, including augmented cell resistance to conventional chemo-/immuno-therapeutics and endogenous immuno-mediated cytotoxicity, as well as invasive behavior and cancer stem cell activities [2].

**Figure 1:**
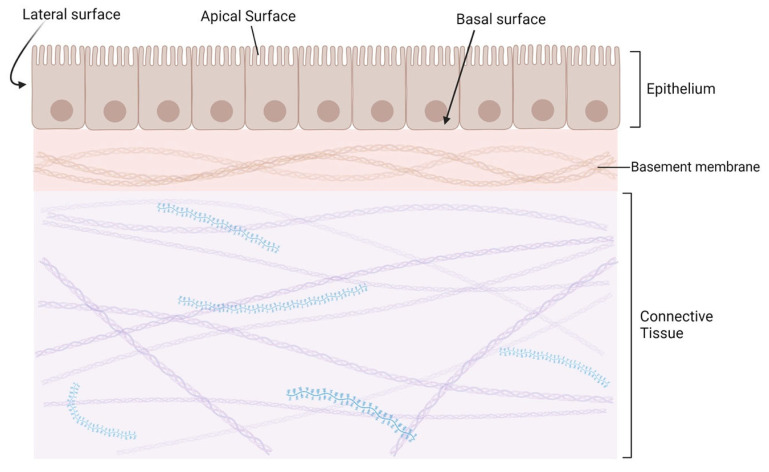
Anatomical structure of epithelial tissue. The epithelium, composed of individual squamous, columnar, or cuboidal shaped epithelial cells, possesses an apical surface, lateral surface, and basal surface. The apical surface faces the lumen, or the external environment, while the basal surface faces the basement membrane. The basement membrane is the layer between the epithelium and the connective tissue found below the membrane. Adapted from “The Extracellular Matrix (ECM)”, by BioRender.com (2024). Retrieved from https://app.biorender.com/biorender-templates (accessed on 6 November 2023).

Epithelial cells are derived from any of the three germ layers, known as the ectoderm, mesoderm, and endoderm [3]. Once formed, the cells link tightly together to create continuous sheets that serve as surfaces like the epidermis and linings, such as those in the digestive tract of the body [3]. Lining epithelia are formed by non-motile apposed cells, held together by various cell junctions, including adherens junctions, desmosomes, and tight junctions that all together contribute to the epithelial integrity and polarity [4]. At the morphological level, the epithelial cells within the epithelium assume a squamous-to-columnar shape and exhibit apical–basal polarity evident in simple epithelia [5]. The apical–basal polarity is critical for epithelial function as it determines the localization of the junction complexes, while it directs the secretion of different molecules to the apical, lateral, or basal membranes of the epithelium [6]. In particular, the basal membrane of non-motile epithelial cells traditionally interacts with the basement membrane [7]. These critical epithelial cell features are illustrated in Figure 1. 

**Figure 2:**
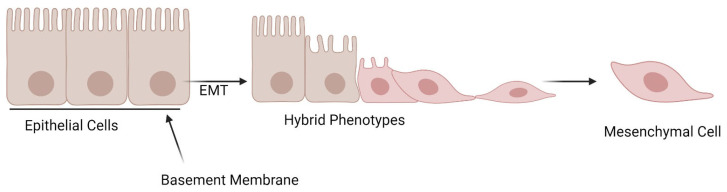
EMT hybrid phenotypes. Traditional epithelial cells, depicted on the left side, are tightly packed together on top of the basement membrane. When these cells undergo EMT, they lose their epithelial traits, such as their cuboidal/columnar shape and tightness, in favor of mesenchymal characteristics, such as motility. EMT functions as a spectrum, enabling epithelial cells to fully convert into mesenchymal cells, or gain only certain traits to resemble hybrids with both mesenchymal and epithelial characteristics, as depicted on the right and middle of the figure, respectively. Adapted from “Epithelial-mesenchymal transition (EMT) of Retinal Pigment Epithelial Cells (RPE)”, by BioRender.com (2024). Retrieved from https://app.biorender.com/biorender-templates (accessed on 6 November 2023).

On the other hand, mesenchymal cells originate from the neural crest and the mesoderm layer during embryonic development, with the earliest known precursor being the mesenchymalangioblast [8,9,10,11,12]. The typical phenotypic features of these cells include the absence of an apical/basolateral polarity, and the absence of stable cell junctions and migratory ability [7,13]. The aforementioned features help mesenchymal cells to define their anatomical structure, as their formation is considered to take place after the degradation of the basement layer under the epithelium, thus enabling them to migrate away from the epithelial layer [7]. In other words, the anatomical structure of mesenchymal cells is starkly opposite to that of epithelial cells. The key differences in anatomy are depicted in Figure 2, which illustrates epithelial cells losing their rigid structure as they undergo EMT. Once the cells have departed from the epithelium layer, their mesenchymal traits facilitate their migration through the extracellular matrix [14].

It has been suggested that the epithelial cells completing the EMT process can travel individually or, if they retain hybrid or epithelial characteristics, along with other cells to new locations using a collective migration [2]. This is quite important, especially during development, where epithelial cells gain the mesenchymal mobility feature for performing their functions at different locations. This is a great example highlighting the close relationship between EMT-related phenotypes and epithelial cells. Furthermore, EMT and epithelial cell cross-talk occurs during wound healing and tissue regeneration, where the epithelial cells undergo EMT by losing their epithelial traits and developing mesenchymal characteristics [5].

The opposing anatomical structures of epithelial and mesenchymal cells are apparent in this process, as the loss of epithelial traits and gain of the mesenchymal phenotype occur hand in hand. The epithelial cells become loosely organized, a mesenchymal characteristic, at the expense of the protective sheets that relied on the tightness of the cells to act as a barrier. The loose organization enables motility, while the epithelial cells also gain invasive properties and greater resistance to programmed cell death through the new mesenchymal phenotype [5]. As previously noted, the extent of EMT is a spectrum, meaning cells can choose to partially adopt the mesenchymal phenotype and obtain only certain traits, or fully express the phenotype, thus gaining all the type-specific traits.

Moreover, the EMT-associated morphological and functional changes that epithelial cells undergo mark a significant step towards the initiation of malignant transformation and cancer cell metastasis [7]. Excessive epithelial cell growth is fundamental to the onset of the neoplastic process, and EMT heightens the risk by strengthening the capabilities of the uncontrolled cell growth. Excessive cell growth and phenotypic changes lead to the gain of traits associated with the mesenchymal phenotype. The enhanced cell mobility and invasiveness, in turn, enable cancer metastasis to take place, as a small number of cancer cells from a primary tumor can activate EMT with intermediate hybrid phenotypes, leading to the acquisition of traits that will facilitate their dissemination from their original site, their entrance into the circulation, and ultimately their spread to other body locations, thus establishing metastases. Noteworthy, collective migration does not need the completion of the EMT program [5]. Acquired resistance to apoptosis and immune evasion can further contribute to the likelihood of cancer cell metastasis to secondary sites as these properties make the elimination of cancer cells much more difficult before their potential spreading [5]. 

This undeniable relationship between EMT and cancer metastasis provides insight into the importance of the Raf Kinase Inhibitory Protein (RKIP), a key regulator of EMT and a metastasis suppressor [15,16]. The function of RKIP was first described as a kinase inhibitor protein by Yeung et al. [15] and has since been recognized as a regulator of many signaling pathways that promote cell survival and growth [17]. In relation to cancer metastasis, RKIP expression gradually diminishes as the aggressiveness of the disease progresses, thus indicating the RKIP loss in tumor cells as a potential driver of metastases’ growth [15,16]. The progressive elimination of RKIP in cancer cells eventually results in a lack of inhibitory proteins necessary to protect signaling pathways from misuse by cancer cells. This lack of tight regulation finally enables uncontrollable cancer cell proliferation, promoting cancer metastasis. 

This review aims to discuss the various properties of the EMT phenotypes and its molecular regulation with emphasis on the hypothesis of the cross-talks between RKIP and selective EMT-inducers SNAIL1/2, TWIST1/2 and ZEB1/2. The various transcriptional and post-transcriptional regulations underlying the expression and activities of these transcription factors (TFs) are presented. We present evidence that our hypothesis was validated and additionally, and has been corroborated by bioinformatic analyses. Hence, we therefore present various strategies to induce RKIP expression in cancer cells in an effort to inhibit the TFs mediating the EMT phenotype and to inhibit tumor growth and metastasis. 

## 2. The EMT Types

According to an early report by Kalluri and Weinberg [7], EMT can be classified into three distinct subtypes defined by functional features, characteristic of different cell growth phases. Specifically, type 1 occurs at various stages during embryonic development, type 2 takes place during periods of tissue regeneration and wound healing, and type 3 is associated with uncontrolled cellular growth. 

### 2.1. Type 1 EMT

Type 1 EMT is associated with the development of the embryo and organ formation, without provoking fibrosis or an invasive cellular phenotype [7,18]. During the gastrulation period, type 1 EMT is required for the epithelial epiblast-derived cells to transit into mesenchymal cells that will later form the three germ layers [19,20]. Another distinction of type 1 EMT is the outcome of mesenchymal cells, as they are the ultimate product of this type, unlike the other two types that result in different cell phenotypes carrying mesenchymal features. After EMT is activated, the cells separate from the epiblast layer and migrate to specific regions within the embryo to form a primitive streak that later results in the germ layers [5,21,22,23]. Hay [21] was the first to describe the connection between EMT and the development of a primitive streak using a chick model. The importance of type 1 EMT is highlighted in the gastrulation process, as traditional epithelial cells are non-motile and tightly packed together, thereby unable to detach from the epiblast layer and travel to specific locations. After EMT occurs, the newly transitioned mesenchymal cells possess the traits to develop the primitive streak observed in reported experiments. Type 1 EMT is activated at the beginning of gastrulation when the fibroblast growth factor (FGF) signaling activates EMT transcription factors, especially Snail1, thus resulting in the transcriptional repression of the epithelial marker E-cadherin, and EMT activation [20,24]. There are several similar dynamic processes during development that utilize type 1 EMT, including neural crest cell migration, somitogenesis, and cardiac valve formation [20,25,26,27].

### 2.2. Type 2 EMT

Type 2 EMT is activated in response to inflammation and is associated with tissue regeneration, wound healing, and fibrosis [5]. The basic paradigm of type 2 EMT involves a repair-associated process where epithelial cells differentiate into fibroblasts by undergoing EMT, thus enabling the rebuilding of damaged tissue following traumatic or inflammatory events [7,18]. Type 2 EMT is induced by a similar mechanism as that of type 1 EMT, starting with expression of EMT inducers, including the SNAIL family of transcription factors, which mediate the suppression of the epithelial markers [5]. While type 1 EMT results in converted mesenchymal cells, type 2 in adults results in fibroblast formation, a cell type that is critical for the development of connective tissue [28]. Fibroblast formation is a hallmark of the wound-healing process, distinguishing type 2 EMT from the other EMT types. In addition, cellular invasion, characteristic of type 3 EMT, does not occur in type 2, further differentiating the EMT types.

Specifically, during wound healing, keratinocytes at the border of the wound undergo EMT to obtain a metastable state, allowing them to move while remaining in contact with their surroundings [5,14]. Keratinocytes reflect the efficiency of the EMT spectrum by acting as hybrids; on the one hand, they remain in contact with the surroundings as epithelial cells, and, on the other hand, they possess the mesenchymal phenotypic trait of motility. Regarding tissue fibrosis, myofibroblast buildup and large amounts of secreted collagen eventually compromise organ function, thus leading to organ failure [5,14,29]. In this case, epithelial cells undergo type 2 EMT to become myofibroblasts, which reduce wound size during healing periods. However, the continued myofibroblast presence increases the risk of organ failure. 

Hence, EMT types 1 and 2 differ in their respective responses to the environments of epithelial cells. EMT type 1 is activated when epithelial cells require mesenchymal traits to perform their roles during development, while type 2 is only induced during periods of inflammation and healing. The context of the environment at the time of EMT activation further solidifies the need to differentiate between types 1 and 2. If cells undergo type 2 EMT during embryogenesis, it will likely result in a lethal outcome due to improper development of tissue layers or a general buildup of myofibroblasts, which eventually lead to organ failure.

### 2.3. Type 3 EMT 

Type 3 EMT occurs in carcinogenesis that has previously undergone genetic and epigenetic modifications, specifically in genes that increase the formation of localized tumors [7,18]. Examples of such modified genes include oncogene and tumor suppressor genes. The presence of modified neoplastic cells with an invasive phenotype is unique to type 3 EMT, differentiating it from the other two EMT types. The functional distinction of type 3 EMT lies in causing further damage to the microenvironment, unlike types 1 and 2 EMT, which are involved in fundamental processes. For example, cells undergoing type 3 EMT acquire the mesenchymal phenotypic traits of motility, invasiveness, and increased resistance to apoptosis that allow for their detachment from their original location and their release into the bloodstream [5,30,31,32]. Using circulation as a means of travel, a small number of tumor cells spread to distant locations, evade apoptosis with their enhanced resistance and expand further in metastatic sites. 

The functional distinction of type 3 EMT offers valuable opportunities towards improving cancer treatments, as cancer metastasis is partially influenced by this EMT type, which, in turn, complicates treatment efficacies. Potential avenues of cancer treatment can utilize EMT regulators to prevent neoplastic cells from gaining increased motility and resistance to apoptosis, thus accelerating treatment effectiveness. Phenotypically, cancer cells undergoing type 3 EMT express either a combination of epithelial and mesenchymal markers, or present a fully mesenchymal phenotype, thus supporting the notion that EMT operates as a spectrum [5,14,33,34]. The existence of a hybrid phenotype provides a major advantage for cancer cells, as it enables them to possess the beneficial qualities of both epithelial and mesenchymal traits. 

## 3. Epithelial and Mesenchymal Biomarkers

### 3.1. Epithelial Markers

Epithelial and mesenchymal cell phenotypes are distinguished according to their respective biomarkers. The major biomarkers associated with epithelial cells include E-cadherin (CDH1 gene), claudins, occludins, zona-occludens 1 (ZO-1), and Mucin-1 (MUC1) [5,35]. E-cadherin, an adhesion molecule of adherens junctions, is considered one of the most common epithelial markers as it contributes to the tightness and non-motility that defines the epithelium. Claudins, occludins, and ZO-1 are key components of tight junctions that impact the tightness of epithelial cells in the epithelium, while MUC1 is a gene that promotes the formation of a mucus barrier to protect epithelial cells [5,35]. 

### 3.2. Mesenchymal Markers

Significant biomarkers for mesenchymal cells include N-cadherin (CDH2 gene), vimentin, fibronectin (FBN), and fibroblast-specific protein 1 (FSP1) [5]. CDH2 is a transmembrane protein that facilitates cell–cell adhesion and mediates the activation of the mitogen-activated protein kinase (MAP K), extracellular signal-regulated kinases (ERK) and phosphoinositide-3-kinase (PI3K) signaling cascades [36]. Vimentin is an intermediate filament protein that helps maintain the structural integrity of mesenchymal cells, while FBN, a glycoprotein secreted and located in extracellular matrix, facilitates communication between the intracellular and extracellular environments. Given that EMT functions as a spectrum, phenotypic hybrid cells can display either a variety of epithelial and mesenchymal biomarkers or complete mesenchymal biomarkers if fully converted. These shared biomarkers are summarized in Table 1. 

Table 1 summarizes the common biomarkers expressed in epithelial and mesenchymal cells. The biomarkers help to clarify if an epithelial cell has undergone EMT because there will be a decrease in epithelial biomarkers and increased expression of the mesenchymal biomarkers. Depending on the extent of transition, the cells can display full mesenchymal biomarkers or a hybrid between the two.

### 3.3. Differentiation of EMT Types

While EMT types can be identified by their context, distinguishing them by specific biomarker expression is complicated as there are no unique markers for each type. Instead, a combination of biomarkers is required to differentiate them. After type 1 EMT takes place, cells can acquire cell-surface proteins, including N-cadherin, alpha-5-beta-1 (*α*5*β*1) integrin, alpha-V-beta-6 (*α*V*β*6) integrin, syndecan-1, and the cytoskeletal marker vimentin, and extracellular matrix (ECM) proteins like *α*1(I) and *α*1(III) collagens, fibronectin, and laminin 5 [28]. In contrast, the fibroblast-specific protein 1 (FSP1), collagen I, discoidin domain receptor tyrosine kinase 2 (DDR2), vimentin, and desmin are considered major markers for identifying cells undergoing type 2 EMT in response to inflammation [7,42,43,44]. Lastly, cells that have undergone type 3 EMT can express a mixture of markers, such as the cell-surface proteins *α*5*β*1 integrin, *α*V6 integrin, syndecan-1, and the cytoskeletal marker *α*-SMA, and ECM proteins *α*1(I) and *α*1(III) collagens [28]. The varying biomarker panel of each EMT type reflects the complexity of EMT, as there is an overlap in the marker changes during the epithelial cell transition; however, the changes differ slightly due to the functional distinctions among the EMT types.

The acquired cytoskeleton markers expressed in all three EMT types include FSP1 and β- catenin [28,43,45]. FSP1 serves as a marker of fibroblasts in different organs and is observed in all EMT types due to fibroblasts’ prominent role in tissue formation. β-catenin is a protein capable of transducing signals to the nucleus as a member of the wingless-related integration site (Wnt) signaling pathway. 

## 4. Molecular Regulation of EMT

### 4.1. Signaling Pathways Inducing EMT

#### 4.1.1. The TGF-β Signaling 

The most well-known signaling inducer of EMT is the transforming growth factor beta (TGF-β) pathway. TGF-β is involved in many cellular processes, including cell growth, differentiation, and migration. Within the TGF-β superfamily, important isoforms related to EMT include TGF-β1, TGF-β2, TGF-β3, and six isoforms of the bone morphogenetic protein (BMP), specifically BMP2 through BMP7 [46]. TGF-β1 regulates the majority of EMT occurrences, regardless of type, while TGF-β2 regulates type 1 EMT during heart development and TGF-β3 regulates type 1 EMT in palate formation [46,47,48,49]. TGF-β1 will be the primary focus of discussion, as it is involved in all EMT types, including type 3 events related to cancer metastasis. Regarding the BMP isoforms, studies have shown that BMP2 and BMP4 activate EMT in malignant cells, while BMP7 counteracts EMT in breast cancer cells [46,50,51,52,53]. BMP3, BMP5, and BMP6 are not involved in type 3 EMT events.

The TGF-β signaling involves the TGF-β ligand itself and a heterotetrametric receptor complex composed of type 1 (TGF-βR1) and type 2 (TGF-βRII) TGF-β receptors. Upon ligand binding to the TGF-βRII receptor, TGF-BR1 is recruited to the complex and is subsequently phosphorylated by TGF-BRII [54]. Once phosphorylated, TGF-βR1 serves as a signaling molecule that triggers other signaling cascades from the surface of epithelial cells. Similarly, BMP signaling operates through receptor stimulation by BMP ligands, with a type II BMP receptor replacing TGF-βRII, and a type I BMP receptor being phosphorylated. 

#### 4.1.2. The RTK Signaling

Another signaling pathway in EMT is the Receptor Tyrosine Kinase (RTK) signaling pathway. RTK can be activated by various growth factors (GF), including the epidermal growth factor (EGF), fibroblast growth factor (FGF), insulin growth factor (IGF), and platelet-derived growth factor (PDGF) [46,55,56,57,58]. Upon ligand binding to RTK, it causes receptor dimerization and trans-phosphorylation of its intracellular domains [46,59]. Receptor dimerization is necessary to induce its kinase activity, which subsequently activates intracellular signaling cascades. Both RTK and TGF-β share the concept of a molecule being phosphorylated, thus allowing a signal to be released and culminating in a cascading effect. 

The constitutive or aberrant activation of RTK results in increased proliferation, survival, and metastasis. The regulation of EMT is centered on several signaling pathways, including TGF-β, NOTCH, Wnt, TNF-α, Hedgehog, and RTKs. Those pathways converge on the transcription factors, such as SNAIL1, SLUG, TWIST1/2, and ZEB1/2 [60]. The RTK signaling pathways downstream activate C-jun, which regulates the EMT transcription factors. 

#### 4.1.3. The Wnt Signaling 

Another major signaling pathway in EMT is the Wnt cascade. Wnt signals traverse the plasma membrane via the Frizzled, a family of G protein-coupled receptors, and lipoprotein receptor-related protein (LRP) receptors [46]. When Wnt signaling is inactive, β-catenin is phosphorylated by glycogen synthase kinase-3 beta (GSK-3β) and sequestered in the cell’s cytoplasm, awaiting degradation [46,61]. Upon Wnt ligand binding to Frizzled or LRP, GSK-3β becomes unable to phosphorylate β-catenin, thus allowing it to transduce a signal to the nucleus. Upon β-catenin-mediated signal transduction to the nucleus, the transcription of *CDH1* gene is inhibited, leading to E-cadherin deficiency, which, in turn, signals the loss of the epithelial cell traits.

### 4.2. EMT-Associated Transcription Factors 

The intracellular signal transduction following the activation of the EMT-inducing pathways Wnt, TGF-β and RTK ultimately results in the activation of EMT transcription factors. The six major transcription factors (TF) of EMT include zinc-finger binding transcription factors SNAIL1 and SNAIL2 (SLUG), as well as zinc finger E-box-binding homeobox 1 (ZEB1), ZEB2, TWIST1, and TWIST2 [5,46,62,63]. SNAIL1 and SNAIL2 are members of the SNAIL superfamily of proteins, while ZEB1, ZEB2, TWIST1, and TWIST2 are basic helix–loop–helix (bHLH) factors. Once activated, these TFs play critical roles in the transcriptional repression of epithelial characteristics and the promotion of mesenchymal traits. SNAIL1 and SNAIL2 act as transcriptional repressors by binding to and repressing the transcription of *CDH1*, which encodes for E-cadherin. E-cadherins serve as the major epithelial biomarkers as they contribute to the adherens junctions that characterize the epithelial cells. While E-cadherin downregulation is an important EMT marker, it was demonstrated that cancer cells with hybrid phenotypes and undergoing collective migration retain high levels of E-cadherin that is upregulated to favor the invasive process [64]. 

In general, these transcription factors reduce the expression of several epithelial markers, including E-cadherin, claudin, occludin, and ZO-1, thereby removing the defining traits that maintain the integrity of the epithelium, such as tight junctions and non-motility. 

## 5. Regulation of EMT TFs (SNAIL1/SNAIL2, TWIST1/TWIST2, ZEB1/ZEB2)

### 5.1. SNAIL Superfamily of TFs 

SNAIL1 and SNAIL2 are members of the SNAIL superfamily of TFs, and these play a major role in EMT induction. SNAIL proteins share a similar structure of a highly conserved C-terminal domain consisting of four to six C2H2 type zinc fingers that can bind to the E-box motif of target gene promoters [65,66]. On the other hand, all vertebrae SNAIL proteins include a conserved SNAG domain in their respective N-terminal that enables SNAIL binding to transcriptional co-repressor complexes [66]. SNAIL proteins also have a serine-rich domain (SRD) and a nuclear export sequence (NES) in their main region that help in protein stabilization [66,67].

On a biological level, activated SNAIL1 and SNAIL2 inhibit E-cadherin expression by directly binding to its promoter at E-box sequences [68,69]. Once bound, SNAIL1 and SNAIL2 repress transcription by recruiting the polycomb repressor complex (PRC), which includes G9a, methyltransferases enhancer of zeste homolog 2 (EZH2), histone deacetylases 1, 2, and the Lys-specific demethylase 1 (LSD1) [70,71]. The components of PRC facilitate histone hypermethylation and deacetylation to inhibit epithelial gene expression [70,71,72,73,74]. Histone hypermethylation and deacetylation are significant because they actively decrease gene expression levels. In this case, PRC activation results in decreased expression of the *CDH1* gene that encodes E-cadherins. 

After SNAIL binding to the E-cadherin promoter, transcription is blocked, causing epithelial cells to lose their vital gap junctions, which is the first step towards acquiring mesenchymal characteristics. Studies have shown that *SNAIL* genes tend to be overexpressed in various cancer types, including SNAIL1 and SNAIL2 upregulation in ovarian and colorectal cancers, respectively [69,75,76,77]. This overexpression is logical given the connection between EMT and cancer metastasis, as cancerous cells, in order to metastasize, must lose their epithelial features and obtain mesenchymal traits, which will eventually allow them to migrate to distant regions of the body.

#### 5.1.1. Transcriptional Regulation of SNAIL Expression

As previously mentioned, multiple signaling pathways, including TGF-β, RTK, and Wnt, are directly and/or indirectly involved in the transcriptional regulation of *SNAIL*. RTK signaling is activated by the hepatocyte growth factor (HGF), fibroblast growth factor (FGF), or epidermal growth factor (EGF), and utilizes the RAS-MAPK pathway to induce SNAIL expression [66,78,79]. Lu et al. showed that EGF overexpression in A431 cells resulted in the downregulation of caveolin-1 and subsequent SNAIL upregulation [79] in a MAPK-dependent manner [68,80,81]. 

SNAIL transcription is further regulated by the TGF-β signaling pathway through the contribution of small mother against decapentaplegic (SMAD) proteins. The activation of TGF-β signaling results in the formation of heterotetrametric complexes involving type I and type II serine/threonine kinase receptors [82]. Upon complex formation, the type II receptor phosphorylates the type I, thus activating it and enabling the phosphorylation of downstream targets especially SMADs [82,83,84]. 

The SMAD family consists of three major subtypes: receptor-activated (R) SMADs, which include SMAD2 and SMAD3 targeted by TGF-β, and SMAD1, SMAD5, and SMAD8 targeted by BMP receptors; a common mediator SMAD (SMAD4); and two inhibitory SMADs (SMAD6 and SMAD7). The type I receptor in the TGF-β pathway phosphorylates R-SMADs at their C-terminal, causing the formation of oligomeric complexes between R-SMADs and SMAD4. These complexes can then translocate to the nucleus to regulate specific gene expressions [82,85]. Oligomeric SMAD complexes function with high-mobility group A2 (HMGA2) to cooperatively bind to the promoter of the SNAIL gene, thereby inducing SNAIL expression [66,86]. Specifically, SMAD complexes collaborate with HMGA2 to induce SNAIL1 expression, while complexes with myocardin-related transcription factor A (MRTF) induce the expression of SNAIL2 [82].

Beyond the TGF-β and SMAD signaling regulations, TGF-β cooperates with the NOTCH and Wnt signaling pathways to regulate SNAIL expression. In NOTCH signaling, there are four known NOTCH receptors (NOTCH 1–4) and two ligand families (Delta/Delta-like and Serrate/Jagged). The binding of a ligand to a Notch receptor triggers two proteolytic cleavage events that release the NOTCH intracellular domain (NIC) [66,87]. The NIC then enters the nucleus and binds to the transcription factor RBPJK/CBF1, converting it from a repressor to an activator to induce target genes [87]. Jagged1 and HEY1 are upregulated through NOTCH activity and promote the expression of SLUG [66,88]. NOTCH regulates SNAIL expression both directly and indirectly: directly by transcriptional activation of SNAIL through Jagged1 and HEY1, and indirectly through the lysyl oxidase (LOX) [66]. NOTCH signaling recruits hypoxia-inducible factor-1α (HIF-1α) to the promoter of LOX, increasing its expression and leading to the stabilization of the SNAIL protein [66,89,90].

Wnt signaling regulates SNAIL expression by modulating the activity of glycogen synthase kinase-3β (GSK-3β). When Wnt signaling is active, GSK-3β is inhibited by phosphorylation and, in turn, cannot phosphorylate its main targets, β-catenin and SNAIL. As a result, both targets are stabilized and accumulate in the nucleus [66]. Conversely, when Wnt signaling is inactive, GSK-3β phosphorylates SNAIL1, tagging it for nuclear export and degradation, thus decreasing its expression [69,91]. Overall, GSK-3β directly regulates SNAIL expression by inducing its phosphorylation when Wnt is inactive. This regulation has a clear connection to EMT, as GSK-3β also phosphorylates β-catenin, a major mesenchymal biomarker. When Wnt signaling is active and GSK-3β is inhibited, β-catenin is not phosphorylated, enabling it to transduce signals to the nucleus, which eventually suppresses *CDH1* transcription and promotes EMT. 

The NF-κB signaling pathway is another mechanism that regulates SNAIL expression through both transcriptional and post-translational mechanisms. Studies have indicated that SNAIL expression can be directly activated in Drosophila via the NF-κB homologue Dorsal [66,92]. Similarly, in humans, NF-κB binds to the SNAIL promoter in the region around -194 and -78, increasing SNAIL transcription [66,93]. NF-κB signaling can be activated by Protein Kinase B (Akt) through phosphorylation of IKKα, leading to SNAIL upregulation [66,94,95,96,97]. The stabilization of SNAIL begins with the transcription of COP9 signalosome 2 (CSN2), which prevents the phosphorylation and ubiquitylation of SNAIL by blocking the interaction between SNAILl and GSK-3β [66,98]. SΝAΙL is further stabilized by the combined effects of the NF-κB pathway and tumor necrosis factor alpha (TNF-α), a major inflammatory cytokine known to stabilize SNAIL [66,98,99]. This intricate regulation underscores the significant role of NF-κB in maintaining SNAIL expression and stability, linking it to the broader processes of inflammation and cancer progression.

#### 5.1.2. Post-Translational Regulation of SNAIL Expression

Both SNAIL1 and SNAIL2 are extremely unstable proteins with a half-life of approximately one hour due to rapid proteasomal degradation [100,101,102]. SNAILl stability, localization, and expression can be regulated via phosphorylation, ubiquitination, and lysine oxidation. Studies have shown that SNAIL can be subjected to proteasomal degradation after GSK-3β-dependent phosphorylation at two motifs within the SNAIL protein (motif 1 and motif 2), followed by β-TrCP-mediated ubiquitination [66,103]. GSK-3β initially binds and phosphorylates SNAILl at motif 2, causing a conformational change that prompts nuclear export. Subsequent phosphorylation of motif 1 by GSK-3β facilitates the interaction between SNAIL and β-Trcp, leading to SNAIL degradation in the cytoplasm [66,103].

Other post-transcriptional regulations of SNAIL include GSK-3β-independent mechanisms. Unlike GSK-3β, p21-activated kinase (PAK1) phosphorylates SNAIL1 at Ser246, tagging it for nuclear localization and increasing its retention and expression in the nucleus [66,69,104]. Additionally, the Small C-terminal domain phosphatase (SCP) can dephosphorylate SNAIL proteins to enhance SNAIL activity [69,105]. SNAIL can also be stabilized through hyperglycemia-regulated O-linked B-N-acetylglucosamine (O-GlcNAc) modification at Ser112, which increases its repressor role by preventing GSK-3β-mediated phosphorylation [66,106]. Lysyl Oxidase Like 2 (LOXL2) further stabilizes SNAIL by inhibiting the binding of F-box and leucine-rich repeat protein 4 (FBXL14) to SNAIL, which otherwise leads to its ubiquitination and degradation [66,107,108]. In the absence of LOXL2, FBXL14 can interact with either phosphorylated or dephosphorylated SNAIL to promote its degradation [66,108]. 

As previously discussed, the TNF-α association with NF-κB signaling induces CSN2, which prevents the binding of GSK-3β and β-Trcp to SNAIL, thereby stabilizing SNAIL by inhibiting its phosphorylation and ultimately its degradation [66,99]. Additionally, the large tumor suppressor kinase 2 (Lats2) directly phosphorylates SNAIL at Thr203, enhancing its stability in the nucleus [66,109]. Finally, phosphorylation of SNAIL by cAMP-activated kinase Protein Kinase A (PKA) and the ubiquitous serine/threonine protein kinase CK2, at Ser11 and Ser92, respectively, results in the upregulation of SNAIL [66,110]. 

These various mechanisms collectively contribute to the complex regulation of SNAIL expression and stability, impacting processes such as EMT and cancer progression.

#### 5.1.3. Post-Transcriptional Regulation of SNAIL Expression

Epigenetic regulation of SNAIL is mainly mediated by small non-coding RNAs (miRNAs) that bind to mRNA to prevent translation or promote mRNA degradation [1,111]. Known miRNAs that inhibit SNAIL1 expression are the miRNA-29b and miRNA-30a, while SNAIL2 is repressed by miRNA-1 and mrRNA-200b [111,112,113,114]. 

### 5.2. TWIST Family of TFs 

TWIST1 and TWIST2 are classified into the family of basic helix–loop–helix (bHLH) transcription factors. Both TWIST proteins form heterodimers or homodimers with isoforms of the E2A gene, E12 and E47, to bind to E-box DNA sites [71,115,116]. The dimer complexes specifically bind to the sequence 5’-CANNTG-3’ found in the E-box, thus enabling TWIST TFs to function either as activators or repressors of gene transcription [115,116]. The ability of TWIST proteins to both inhibit and promote transcription is crucial in the EMT process, where they repress epithelial gene transcription and promote transcriptional activation of mesenchymal genes. Studies have shown that increased expression of TWIST1 and TWIST2 leads to decreased expression of E-cadherins and increased expression of the mesenchymal markers Fibronectin, N-cadherin, and Vimentin [69,117,118]. 

A study by Casas et al. further highlighted a significant relationship between TWIST proteins and SNAIL2, demonstrating that TWIST proteins could not activate EMT in mammary cells when SNAIL2 was knocked out [71,119]. This suggests that TWIST proteins may not directly repress the transcription of E-cadherin, but rather act indirectly to inhibit transcription of epithelial cell genes. This indirect mechanism could involve the regulation of other transcription factors or co-factors necessary for the repression of epithelial markers and the promotion of mesenchymal traits.

#### 5.2.1. Epigenetic Regulation of TWIST TFs

TWIST proteins can be regulated via epigenetic mechanisms, including methylation. Multiple myeloma SET domain (MMSET) was shown to directly activate TWIST1 expression by binding to its promoter and increasing methylation at H3K36m2 [120,121]. This specific methylation mark is associated with active transcription and facilitates the expression of TWIST1 by altering the chromatin structure to a more open and accessible state [120]. 

#### 5.2.2. Transcriptional Regulation of TWIST TFs

Transcriptional regulation of TWIST proteins occurs under hypoxic conditions, where tissue oxygen levels are insufficient. Previous studies have shown that HIF-1α, a subunit of the hypoxia-inducible factor (HIF)-1 complex, is upregulated in solid tumors under hypoxic conditions [122,123]. In response to hypoxia, HIF-1α translocates from the cytoplasm to the nucleus, where it dimerizes with HIF-1β, forming the HIF-1 complex. This complex then activates downstream genes associated with cancer progression [123,124]. Yang et al. [123] utilized cells from the FTC133 cell line to study the significance of HIF-1α and discovered a proportional relationship between HIF-1α and TWIST TFs. Overexpression of HIF-1α led to increased TWIST expression, while suppression of HIF-1α reduced TWIST expression [123].

Another mechanism regulating TWIST TFs involves the NF-κB signaling pathway. A study by Šošić et al. [125] explored whether TWIST1 and TWIST2 are regulated by TNF-α, an activator of the NF-κB pathway. Inducing TNF-α in immortalized mouse fibroblasts resulted in increased expression levels of TWIST1 and TWIST2 [125]. The study further indicated that this upregulation was dependent on NF-κB signaling, confirming a relationship between TWIST TFs and the NF-κB pathway [125].

These findings highlight the complex regulatory mechanisms of TWIST TFs, involving both hypoxia-induced and NF-κB signaling pathways, which contribute to the transcriptional regulation crucial for cancer progression and EMT.

#### 5.2.3. Post-Transcriptional Regulation of TWIST TFs

TWIST transcription factors can be regulated post-transcriptionally via numerous microRNAs (miRNAs). A study conducted by Wu et al. showed that ectopic expression of miRNA-532 in the cystic fibrosis pancreatic adenocarcinoma cell line (CFPAC-1) resulted in reduced TWIST1 mRNA and protein levels [126]. The specificity of the findings was further validated by the upregulation of TWIST1 mRNA expression following miR-532 knocking down or cell treatment with a miR-532 inhibitor [126]. 

A second regulatory miRNA of TWIST1 is miRNA-720, which directly targets TWIST1 and exhibits an inverse relationship with it [127,128]. In a study by Li et al., luciferase reporter assays in human embryonic kidney 293T cells (HEK-293T cells) showed an upregulation of TWIST1 protein following miRNA-720 inhibition and a decrease in TWIST1 levels after miRNA-720 overexpression [127,128]. A similar inverse relationship was observed in human breast cancer tissues, where a gradual decline in miRNA-720 correlated with increased TWIST1 protein levels [127,128]. 

#### 5.2.4. Post-Translational Regulation of TWIST TFs

Phosphorylation serves as a crucial post-translational regulator of TWIST TFs. Hong et al. identified serine 68 (S68) as a significant phosphorylation site on TWIST1 and observed a positive relationship between S68 phosphorylation and TWIST stability [69,129]. The study showed that phosphorylation of S68 through the P38, JNK, and ERK1/2 signaling pathways increased TWIST1 protein levels [129]. Moreover, MAPK signaling, activated by Ras or TGF-β signaling, further upregulated S68 phosphorylation and TWIST1 expression [129]. Conversely, when Hong et al. introduced an alanine mutation at S68 (S68A), it significantly accelerated TWIST1 degradation [129]. 

These regulatory mechanisms highlight the complex layers of control over TWIST1, involving both miRNA-mediated and phosphorylation-dependent pathways, which are crucial for modulating its role in processes such as cancer progression and EMT.

### 5.3. ZEB Family of TFs 

The zinc-finger E-box binding homeobox (ZEB) family in humans includes ZEB1 and ZEB2, which serve as transcription factors of EMT. Once ZEB1 and ZEB2 are activated, the proteins use their zinc-finger regions to bind at the Enhancer-box (E-box) of gene sequences, resulting in reduced transcription of epithelial markers and increased expression of mesenchymal characteristics [71,130]. E-boxes typically enhance gene transcription by activating or increasing the transcriptional activity of associated genes. This activity depends on the binding of specific TFs, such as ZEB1 and ZEB2 in the context of EMT. Thus, ZEB TFs likely bind to E-boxes of mesenchymal genes to enhance their transcriptional activity while avoiding E-boxes associated with epithelial genes. 

Studies have demonstrated that ZEB transcription factors (TFs) repress gene transcription through multiple mechanisms. One such mechanism involves the recruitment of the corepressor C-terminal-binding protein (CTBP) [71,131,132]. Additionally, ZEB TFs interact with the switch/sucrose nonfermentable (SWI/SNF) chromatin-remodeling protein Brahma-related gene 1 (BRG1) in certain cancer cells to suppress gene transcription [71,133]. Furthermore, ZEB1 can switch from acting as a repressor to functioning as a transcriptional activator by interacting with the histone acetyltransferase P300/CBP-associated factor (PCAF) [71,134]. Both ZEB1 and ZEB2 are associated with increased expression of Vimentin and N-cadherin, which are key markers of mesenchymal traits [71,135]. 

#### 5.3.1. Epigenetic Regulation of ZEB TFs

The transcriptional status of ZEB1 is largely controlled through post-translational modifications of the histones associated with ZEB1. The transcription of ZEB1 is activated via trimethylation of lysine 4 on the histone H3 subunit (H3K4me3), while elongation is related to dimethylation of lysine 79 on the histone H3 subunit (H3K79me2) [136,137,138]. A high presence of H3K4me3 and H3K79me2 suggests a gene is being actively transcribed, while trimethylation of lysine 27 on the histone H3 subunit (H3K27me3) is associated with transcriptional repression of specific genes [138,139]. Both H3K4me3 and the repressive H3K27me3 occupy the bivalent chromatin domain, a chromatin region associated with the promoter of ZEB1 [138,140]. The bivalent domain inhibits ZEB1 transcription, but the overall transcriptional status can be switched to either inhibit or promote ZEB1 expression [138]. Accordingly, a previous study demonstrated that high transcriptional levels of ZEB1 were found in the presence of high levels of H2K3me3 and H3K79me2, while H3K4me3 was absent from the promoter of ZEB1 [138,141]. 

#### 5.3.2. Transcriptional Regulation of ZEB TFs

The TGF-β signaling pathway activates ZEB1 transcription by increasing SMAD2 expression, which, in turn, forms heterotrimeric complexes with SMAD4 to activate the transcription of target genes [138,142,143]. SMAD2 expression is induced by various TGF-β signaling molecules, including TGF-β1 and TGF-β2 [144]. Inhibition of TGF-β1 was shown to decrease ZEB mRNA levels in Madin-Darby Canine Kidney (MDCK) cells, thus highlighting the significance of TGF-β1 in this regulatory process [144]. Additionally, research by Nomura et al. demonstrated that the inhibition of NF-κB signaling results in decreased ZEB1, while active NF-κB signaling increases ZEB1 levels [138,145].

ZEB expression is uniquely regulated by other major EMT TFs, like TWIST1 and SNAIL1 [1,146]. Dave and colleagues found that TWIST1 cooperates with SNAIL1 to induce ZEB1 expression, as evidenced by the decreased levels of ZEB protein when both SNAIL1 and TWIST1 were knocked out [1,146].

#### 5.3.3. Post-Transcriptional Regulation of ZEB TFs

The expressions of ZEB1 and ZEB2 are negatively regulated by the miR-200 family members miR-200a, miR-200b, miR-200c, miR-141, and miR-429, which specifically target ZEB1 and ZEB2 mRNAs [144,147,148,149,150]. The miR-200 family and miR-205 downregulate the expression of ZEB proteins, but their own expression is downregulated by the TGF-β signaling, through the activation of the Akt2 serine/threonine kinase [150,151]. ZEB2 shares a unique relationship with miR-200 members, as it binds to the promoter of miR-200, resulting in its downregulation and creating a negative feedback loop between ZEB2 and miR-200 members [144,147,150]. 

In general, the regulation of ZEB TFs is multifaceted, involving signaling pathways, transcriptional regulation, post-transcriptional mechanisms, and epigenetic modifications.

## 6. Mechanisms of RKIP-Mediated Inhibition of EMT 

RKIP can indirectly regulate the expression of EMT-inducing TFs, such as members of the SNAIL, TWIST and ZEB superfamilies, through its inhibitory action on multiple signaling pathways that are analyzed below. RKIP can also impact the stabilization of epithelial markers including E-cadherins, which is detrimental in cells undergoing collective migration/invasion. 

### 6.1. RKIP-Mediated Inhibition of the MAPK/MEK/ERK Pathway

The ERK signaling pathway is a pivotal coordinator of the EMT. Several EMT-inducing factors activate signaling pathways that converge on ERK to regulate the EMT transcription factors. While EMT can be induced by many EMT upstream molecules (for example, FGF, IGF1, EGF, TGF-β), the signaling changes induced by these molecules often converge on the main EMT regulators, such as ERK and PI3K/AKT. ERK is one of the key mediators of the RAS/RAF growth factor receptors and TGF-β-mediated EMT plays a role in cancer progression and promotion [1,152]. Several transcription factors regulate EMT, such as SNAIL1, SLUG, TWIST1, TWIST2, ZEB1, ZEB2, and the expression of these transcription factors is downstream of the ERK pathway [1,152]. 

**Figure 3:**
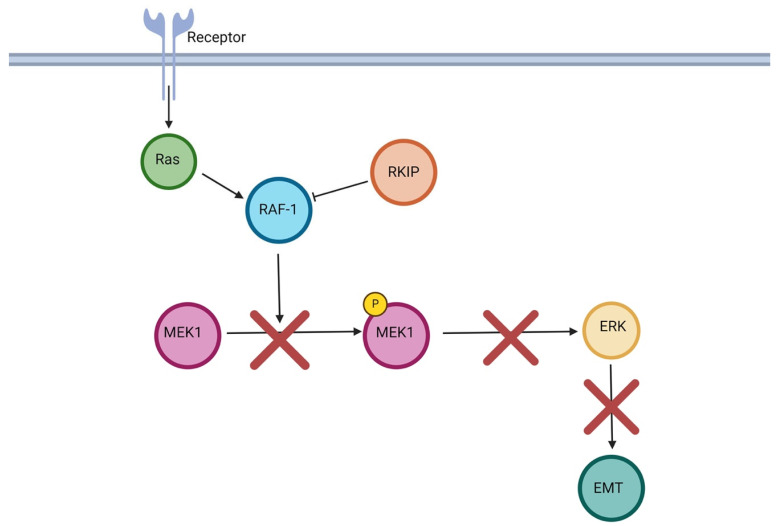
RKIP-mediated inhibition of the RAF/MEK pathway. The activation of RAS through its proper ligand results in the activation of RAF-1 and the MEK/ERK pathway. Once RAF-1 is activated, it phosphorylates MEK-1, resulting in its activation and leading to the activation of ERK. When RKIP is present, it competitively interrupts the interaction between RAF-1 and MEK-1 [15]. As a result, RKIP prevents RAF-1 from phosphorylating MEK-1, ensuring that neither MEK-1 nor ERK are activated. Created with BioRender.com (accessed on 18 April 2024).

RKIP can mediate the inhibition of EMT via its regulatory action on the MAPK/ERK signaling pathway and its downstream targets SNAIL1 and TWIST1. RKIP targets this pathway by inhibiting the interaction between Raf-1 and the mitogen-activated protein kinase 1 MEK1. Briefly, ERKs are activated by MEK1-mediated phosphorylation, which becomes activated when phosphorylated by Raf-1 [15]. Yeung et al. explored the role of RKIP in Raf-1 activity and found RKIP to be a selective inhibitor that blocked Raf-1 from activating MEK1, ultimately leading to decreased activation of ERK/MAPK signaling [15]. Accordingly, RKIP downregulation by antisense vectors or RKIP antibodies resulted in increased ERK/MAPK activation in both cases in Rat-1 cells [15]. These findings indicated that RKIP did not inhibit all phosphorylation activity of the MAPK/ERK pathway; rather, it might primarily regulate MAPK/ERK signaling by interacting with the RAF1/MEK1/MAPK cascade [15]. RKIP-mediated inhibition of the MAPK/MEK/ERK pathway is depicted in Figure 3. 

Yang et al. further found that SNAIL is a downstream effector of the MEK/ERK/MAPK signaling pathway, observing decreased SNAIL expression following the inhibition of ERK signaling [153]. Thus, RKIP directly impacts the expression levels of SNAIL and EMT overall by preventing Raf-1 from interacting with MEK1. This prevention results in an absence of an ERK signal, ultimately disturbing the signaling cascade necessary for SNAIL1 expression. 

### 6.2. RKIP-Mediated Inhibition of NF-κB Activation

RKIP is involved in the regulation of the NF-κB pathway’s activation, which serves as a key signaling system for the expression of several EMT-inducing families of TFs, including SNAIL, ZEB and TWIST. 

NF-κB is primarily found in its inactive form within the cytoplasm, bound to inhibitory proteins called IκB [154,155]. NF-κB signaling is activated following a sequence of phosphorylation events. Upstream kinases like NF-κB-inducing kinase (NIK) and Transforming growth factor beta-activated kinase 1 (TAK1) phosphorylate the IκB kinase complex, IKK, which leads to IκBα phosphorylation, thus resulting in IκB degradation and NF-κΒ translocation to the nucleus [154]. A study conducted by Yeung et al. demonstrated direct physical interaction of RKIP with TAK1 and NIK while also acknowledging a physical association of RKIP with IKK-α and IKK-β [156]. Ectopic expression of RKIP decreased NF-κB activity, while the absence of RKIP expression had the opposite effect on NF-κB activation [156]. These results indicate that RKIP strongly antagonizes NF-κB activation, as it could inhibit the chain of phosphorylation events on IKKs, thus keeping IκBα bound to NF-κB and the latter inactive in the cytoplasm. 

**Figure 4:**
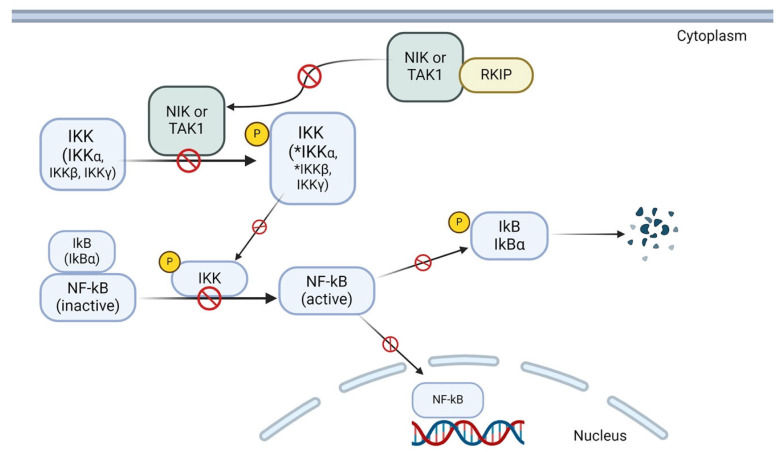
RKIP-mediated inhibition of NF-κB pathway. In the absence of RKIP, NIK or TAK1 are involved in the phosphorylation of the IKK complex, including subunits IKKα and IKKβ. After the IKK complex is phosphorylated, it mediates the phosphorylation of Iκβ, specifically Iκβα, resulting in Iκβ’s degradation while activating and enabling the translocation of NF-κB into the cytoplasm. When present, RKIP physically interacts with NIK and TAK1, preventing the phosphorylation of IKK and indirectly inhibiting the phosphorylation of Iκβ. Thus, when RKIP is present, the NF-κB complex remains attached to Iκβ and inactive, ultimately preventing its translocation into the nucleus. Created with BioRender.com.

Multiple studies have indicated that increased NF-κB activity leads to the upregulation of SNAIL1. RKIP regulates SNAIL1 to some extent by interacting with NIK and TAK1, thereby preventing phosphorylation of IKK and resulting in IκB remaining bound to NF-κB. By preventing the activation of NF-κB, RKIP provides another regulatory mechanism to decrease SNAIL1 transcription and expression. Figure 4 illustrates the general mechanism of RKIP’s involvement in NF-κB regulation, which applies to this section by showing how this regulatory cascade ultimately leads to decreased SNAIL1 transcription.

Accordingly, TWIST and ZEB TFs are also under the transcriptional regulation of the NF-κB pathway. Šošić et al. found increased levels of TWIST1 and TWIST 2 after the treatment of immortalized mouse fibroblasts with TNF-α, an activator of the NF-κΒ signaling [125]. RKIP was shown to reduce TNF-α-mediated NF-κB activation by five-fold in 293 cells via direct interaction with NIK and TAK1 [154], which ultimately results in decreased expression levels of TWIST1 and TWIST2 [156].

### 6.3. RKIP-Mediated Regulation of Other Signaling Cascades 

**Figure 5:**
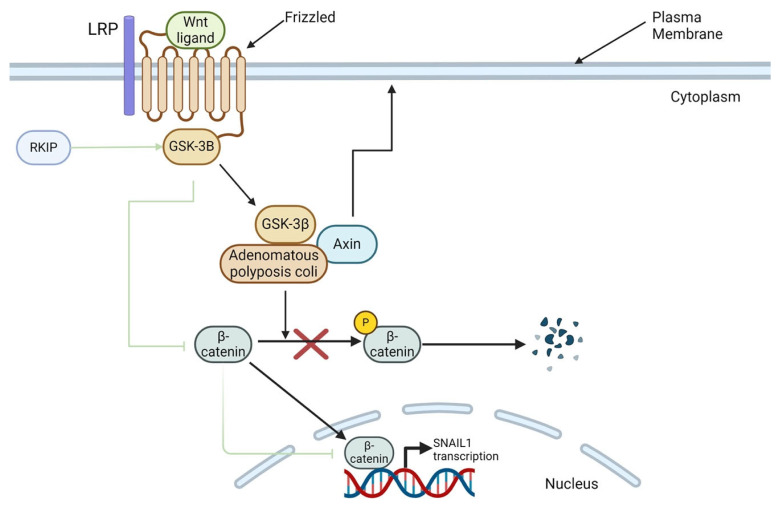
RKIP-mediated inhibition of SNAIL1 via Wnt pathway. When Wnt signaling is absent, a complex composed of GSK-3β, adenomatous polyposis coli, and Axin phosphorylates β-catenin. Once phosphorylated, β-catenin remains in the cytoplasm and is marked for degradation. When a Wnt ligand binds to Frizzled, Axin is recruited towards the plasma membrane, moving away from the complex and ultimately preventing GSK-3β from phosphorylating β-catenin. Thus, when Wnt signaling is active, β-catenin remains unphosphorylated and is translocated into the nucleus, where it upregulates target genes like SNAIL1. RKIP has been shown to increase GSK-3β while decreasing β-catenin, SNAIL1, and SNAIL2 levels, implicating an indirect relationship where RKIP indirectly decreases SNAIL1, and potentially SNAIL2, transcription via upregulation of GSK-3β through the Wnt pathway. Adapted from “GPCR Molecular Pathway (Layout)”, by BioRender.com (2024). Retrieved from https://app.biorender.com/biorender-templates accessed on 9 September 2024.

Wnt signaling activation has also been identified as a key mechanism regulating the transcription of SNAIL1. When Wnt signaling is active, the GSK-3β protein cannot phosphorylate either β-catenin or Snail1, resulting in their stabilization in the nucleus [82,103]. Conversely, when the Wnt pathway is inactive, GSK-3β tags SNAIL1 for nuclear export and degradation, and phosphorylates β-catenin [69,91]. A study by Al-Mulla et al. found that RKIP directly binds to GSK-3β proteins, preventing its inactive phosphorylation, and therefore it is positively correlated with the active form of GSK-3β [157]. To investigate this relationship, Al-Mulla et al. silenced RKIP in HEK-499 cells and observed decreased GSK-3β protein levels. Conversely, RKIP overexpression led to increased GSK-3β protein levels, thus validating the positive association between the two players [157]. Overall, the above findings align with RKIP’s role as a negative regulator of EMT through potential regulation of SNAIL1 and β-catenin stabilization by preventing GSK-3β inactivation. RKIP-mediated inhibition of SNAIL1 via Wnt signaling is depicted below in Figure 5.

**Figure 6:**
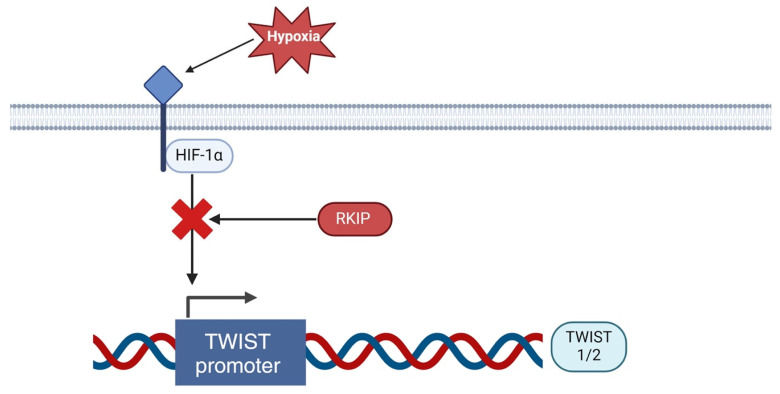
RKIP regulation of TWIST TFs during hypoxia. During conditions of hypoxia, HIF-1α is upregulated and can directly regulate TWIST levels by binding to the hypoxia response element located in the promoter region of the TWIST gene. Periods of hypoxia activate the Raf-1/ERK pathway by increasing the dissociation between RKIP and RAF-1, indicating an inverse relationship between hypoxia/ HIF-1α levels and RKIP. Taking the relationship into consideration, it is possible that RKIP can negatively regulate TWIST TFs by preventing HIF-1α from directly binding to the TWIST promoter. Created with BioRender.com (accessed on 18 April 2024).

RKIP can also regulate TWIST transcription factors (TFs) through its role in regulating NOTCH1 and HIF-1α during hypoxia. Notch signaling is known to recruit HIF-1α to the LOX promoter during hypoxia, and Yang et al. [123] found a positive relationship between the overexpression of HIF-1α and TWIST TFs. The significance of this relationship is highlighted in a study by Noh et al. [158], which found that overexpression of RKIP inhibits NOTCH1 activation. RKIP’s inhibition of NOTCH1 suggests a regulatory mechanism during hypoxia. By decreasing NOTCH1 activation, RKIP reduces HIF-1α expression, which ultimately leads to a decrease in TWIST TFs. The overall scheme of RKIP regulation over TWIST1/2 via inhibited NOTCH1 activation is depicted below in Figure 6.

RKIP further regulates the expression of ZEB transcription factors (TFs) through the TGF-β signaling pathway. ZEB protein levels are proportional to the activity levels of TGF-β1 and TGF-β2 [144]. Previous studies have identified SMAD2 expression, activated by TGF-β signaling, as the major activator for ZEB TFs. RKIP can regulate ZEB TFs by modulating SMAD2 activity.

Palmer et al. [159] further found that YY1 can induce Snail1 transcription, thus highlighting a potential network among these three components. RKIP inhibits the NF-kB pathway and its downstream target YY1. The inhibition of YY1 will result in the inhibition of SNAIL 1. Therefore, this finding shows an indirect mechanism through which RKIP regulates SNAIL 1 expression. 

### 6.4. Role of RKIP in E-Cadherin Stabilization 

**Figure 7:**
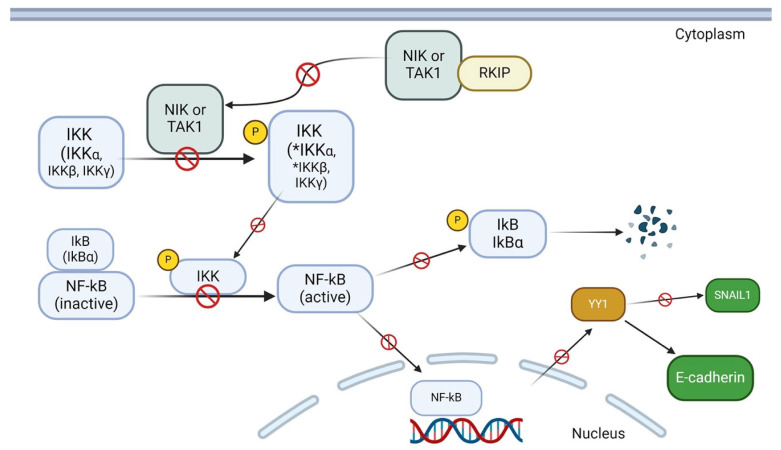
**RKIP-mediated regulation of NF-kB, YY1, and SNAIL1.** When RKIP is absent, NIK or TAK1 are involved in the phosphorylation of the IKK complex, including subunits IKKα and IKKβ. After the IKK complex is phosphorylated, it mediates the phosphorylation of Iκβ, specifically Iκβα, resulting in Iκβ’s degradation while activating and enabling the translocation of NF-κB into the cytoplasm. When present, RKIP physically interacts with NIK and TAK1, preventing the phosphorylation of IKK and indirectly inhibiting the phosphorylation of Iκβ. Thus, when RKIP is present, the NF-κB complex remains attached to Iκβ and inactive, ultimately preventing its translocation into the nucleus. As the NF-kB pathway is known to induce YY1 and SNAIL1 transcription, RKIP-mediated inhibition of NF-kB decreases YY1 and SNAIL1 levels while indirectly stabilizing E-cadherin, a key epithelial marker. Created with BioRender.com (accessed on 18 July 2024).

RKIP contributes to E-cadherin stabilization through an indirect mechanism involving YY1 and SNAIL1. Wang et al. [160] noted that the NF-κB signaling pathway is an upstream inducer of YY1, and Palmer et al. [159] indicated that YY1 can induce SNAIL1 transcription. RKIP is known to inhibit the NF-κB signaling pathway, which results in the inhibition of YY1 and thereby SNAIL1. SNAIL1 is a major transcription factor in epithelial–mesenchymal transition (EMT) and plays a significant role in the loss of E-cadherin expression as cells convert to a mesenchymal phenotype. Therefore, RKIP indirectly contributes to E-cadherin stabilization by hindering the transcription of SNAIL1, thus reducing EMT activity in many of the carcinomas discussed in this paper [74,77,79]. This chain of events is illustrated in Figure 7.

## 7. Regulation of RKIP Expression

### 7.1. Epigenetic Regulation of RKIP 

Research on gastric adenocarcinomas, esophageal squamous cell carcinomas, and breast cancer cells has identified a relationship between methylation of the RKIP promoter and low RKIP expression levels, thus suggesting that RKIP can be epigenetically regulated through promoter methylation [161,162,163,164,165,166,167]. In particular, gastric and esophageal cancers displayed significantly increased rates of hypermethylated RKIP promoters compared to normal mucosal tissue [163,165,166]. 

**Figure 8:**
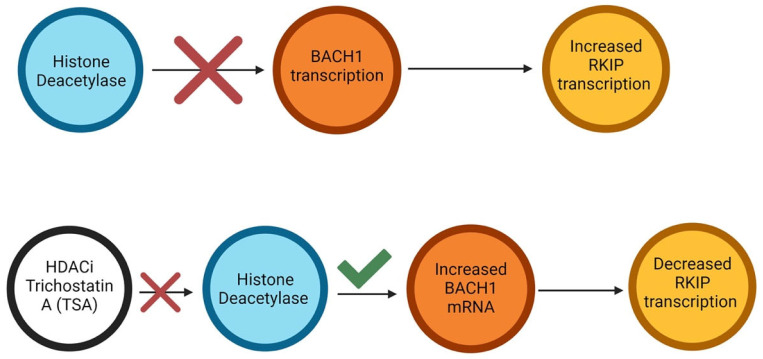
HDAC inhibitors on RKIP expression. The top figure indicates that histone deacetylase results in the acetylation of lysine residues on histones, repressing BACH1 transcription and indirectly increasing RKIP transcription [168]. The bottom figure demonstrates how HDAC inhibitor Trichostatin A (TSA) inhibits histone deacetylase, resulting in increased BACH1 mRNA and leading to decreased RKIP transcription [168]. The two figures indicate an inverse relationship between BACH1 and RKIP transcription in the MDA-MB-231 cell line. Created with BioRender.com (accessed on 7 April 2024).

Lee et al. further identified histone deacetylase inhibitors (HDACi) as potential epigenetic regulators of RKIP via its regulation of the BTB Domain and CNC homolog 1 (BACH1), thus suggesting histone modifications as an additional epigenetic mechanism of RKIP regulation [168]. In particular, exposure of the triple-negative breast cancer (TNBC) cell lines, MDA-MB-231 and 1833, to HDACi trichostatin A (TSA) resulted in time-dependent induction of BACH1 mRNA, therefore demonstrating an inverse relationship between BACH1 and RKIP [168]. TSA inhibits histone deacetylase activity, leading to the acetylation of lysine residues on histones and ultimately activating the transcription of BACH1. As BACH1 serves as a transcriptional regulator of RKIP, HDACi TSA is suggested to act as an indirect inhibitor of RKIP, as shown in Figure 8. 

However, it must be noted that the exact epigenetic mechanisms that regulate RKIP via histone modifications remain largely unclear. For example, Beach et al. performed RKIP immunoblotting and found that HDACi TSA induced a substantial increase in RKIP expression in DU145 prostate cancer cells [169]. Along with the high RKIP levels, the TSA-treated DU145 cells further showed increased apoptosis [169], thus suggesting RKIP involvement in programmed cell death of cancer cells, following HDACi TSA treatment. Labbozzetta et al. reported similar findings, as HDACi TSA-treated cells from the TNBC cell line SUM159 resulted in enhanced RKIP mRNA expression [170]. Contrary to the aforementioned studies, Lee et al. did not observe induced RKIP expression when HDACi TSA was introduced to MDA-MB-231 and 1833 cells [168]. Considering all three studies, it is clear that histone modifications can regulate RKIP expression through complex processes that vary based on different cellular factors 

Another form of epigenetic RKIP regulation is through EZH2, a part of the polycomb repressive complex 2 (PRC2), which catalyzes H3K27me3 and H3K9me3 modifications on the promoter region of RKIP [171]. These modifications result in RKIP transcriptional repression and are dependent on HDAC promoter recruitment [171]. The study found the inhibitory activity of EZH2 was negatively regulated upstream by miRNA-101 [171]. These findings highlight the significance of miRNA-101 in RKIP epigenetic regulation, as it might contribute to RKIP transcriptional activation by inhibiting its epigenetic repression by EZH2. 

### 7.2. Transcriptional Regulation of RKIP Expression 

One transcriptional regulator of RKIP is SNAIL1, one of the main transcription factors of EMT. Beach et al. first demonstrated a negative correlation between SNAIL1 protein expression and RKIP levels in prostate cancer cells [169]. In particular, ectopic expression of SNAIL1 in the non-metastatic cancer cell line LNCaP, known to contain high RKIP levels, resulted in significant downregulation of RKIP [169]. Furthermore, a chromatin immunoprecipitation (ChIP) experiment on LNCaP cells showed that SNAIL1 was associated with the proximal RKIP promoter at E-box 2 of the RKIP gene locus. This association implies that SNAIL1 acts as a direct inhibitor of RKIP transcription and expression [169]. The same study also explored the potential relationship between SNAIL2 (SLUG) and RKIP expression levels. Considering that SLUG shares many physiological functions with SNAIL1 and is part of the SNAIL superfamily of EMT TFs, Beach et al. examined SLUG expression levels in relation to RKIP expression. Interestingly, no statistically significant relationship was established between the studied molecules, thus suggesting that SLUG does not share the inhibitory capability of SNAIL1 on RKIP [169]. 

Another transcriptional regulator of RKIP expression is BACH1. Studies using TNBC cells also found an inverse proportional relationship between BACH1 levels and RKIP expression [166,168,172]. Yun et al. first observed that RKIP inhibits BACH1 levels, while Lee et al. expanded on this relationship by identifying a double-negative feedback loop between BACH1 and RKIP [168,172]. In particular, knocking out BACH1 induced higher RKIP levels, while BACH1 overexpression repressed RKIP expression [168]. Using ChIP assays, Lee et al. further analyzed the direct recruitment of BACH1 to the RKIP promoter and found that BACH1 was significantly recruited to the -373-bp upstream region (RKIP 4) in 1833 cells [168]. The overall results suggest that BACH1 directly binds to the RKIP promoter region and represses RKIP transcription, similarly to how SNAIL1 regulates RKIP.

Another potential transcriptional regulator of RKIP is the transcription factor Yin-Yang 1 (YY1). Previous reports by Trask et al. described YY1’s regulatory role during development [173]. However, YY1’s impact in EMT and uncontrolled cancer growth is less well defined, remaining an area that requires further research. Studies by Bonavida and Baritaki have suggested an inverse relationship between YY1 and RKIP, indicating that YY1 may induce EMT by regulating downstream targets, including RKIP [174,175]. Moreover, a study identified six putative YY1-binding motifs and found direct recruitment of YY1 to only one site on the RKIP promoter in Non-Hodgkin B-cell Lymphoma Ramos cells [176]. After inhibiting YY1 expression in prostate cancer and B-NHL cell lines, full RKIP promoter activity and RKIP protein levels were observed [176]. The findings from the study suggest there might be more transcriptional factors that contribute to reduced RKIP transcription independent of YY1. Overall, YY1 has been proven to play a role in the repression of RKIP expression, but much remains unknown regarding the extent to which YY1 can directly or indirectly reduce RKIP levels [176]. 

Further research has shown that the full RKIP promoter activity is controlled by an interaction between the regions −56 and + 261 relative to the transcription start site [177]. Zhang et al. found that transcription factors Sp1, CREB, and histone acetylase p300 are significant for the RKIP promoter region responsible for full RKIP promoter activity [177]. Knockdown of Sp1, CREB, and p300 in human melanoma and cervical cancer cell lines resulted in decreased RKIP promoter activity, highlighting their potential positive regulatory roles in RKIP transcription [177]. 

**Figure 9:**
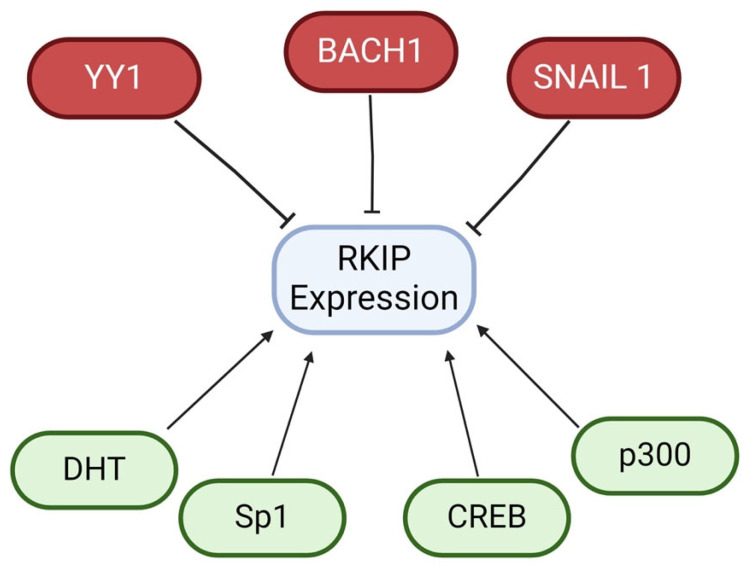
Positive and negative regulators of RKIP expression. YY1, BACH1, and SNAIL1 are negative regulators of RKIP expression on the transcriptional level. On the other hand, DHT, Sp1, CREB, and histone acetylase p300 are positive regulators of RKIP on the transcriptional level. Created with BioRender.com (accessed on 5 June 2024).

Another potential positive regulator of RKIP transcription is androgen dihydrotestosterone (DHT). A study conducted by Zhang et al. found direct binding of the androgen receptor (AR) to a putative androgen responsive element (ARE) found in the RKIP promoter in prostate cells [178]. The results demonstrated that DHT increased RKIP promoter activity and RKIP expression, thus implicating RKIP as an androgen target gene [178]. The positive and negative regulators of RKIP expression are highlighted in Figure 9. 

### 7.3. Post-Transcriptional Regulation of RKIP Expression

**Figure 10:**
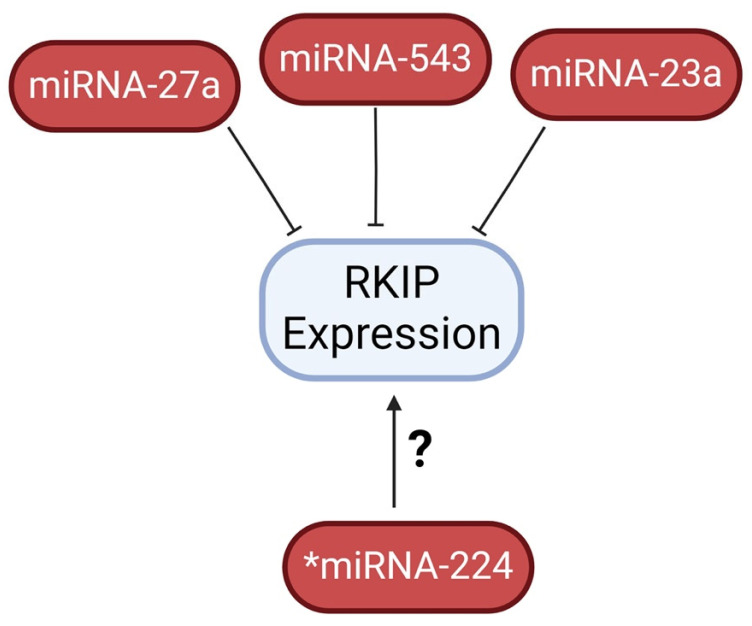
miRNA-mediated regulation of RKIP expression three major miRNAs (miRNA-27a, miRNA-543, miRNA-23a) were found to inhibit RKIP expression. MiRNA-224 contains an asterisk due to the difference in findings between two studies, one finding miRNA-224 to be an RKIP inhibitor, while the second study did not find RKIP to be repressed by miRNA-224 [181,184]. Created with BioRender.com (accessed on 5 June 2024).

RKIP can be post-transcriptionally regulated via multiple miRNAs. Li et al. demonstrated that increased levels of miRNA-27a were associated with low RKIP expression, ultimately identifying miRNA-27a as a direct inhibitor of RKIP in lung adenocarcinomas [179]. Studies on various cancer types, including prostate, breast and AML tumors, further revealed miRNA-543, miRNA-23a, and miRNA-224 also inhibit RKIP translation [180,181,182,183]. Both miRNA-23α and miRNA-224 were shown to bind directly to the RKIP 3’ untranslated region (UTR), while their inhibitions were able to recover RKIP levels. However, Poma et al. failed to show such repression of RKIP by miRNA-224 in hepatocellular carcinoma cell lines [184]. The differing findings between Poma et al. [184] and Hatzl et al. [181] suggest that miRNAs might not act as universal inhibitors for every cancer cell type. I. Additionally, Du et al. found that the tumor suppressor long non-coding RNA (LncRNA) XIST was capable of inhibiting miRNA-23a by direct binding to it and thereby preventing miRNA-23a from inducing degradation of XIST [181]. The various mechanisms of RKIP regulation are illustrated in Figure 10. 

## 8. Clinical Significance of RKIP Expression in Carcinomas

The significance of RKIP expression in carcinomas has been described in multiple studies [185,186,187,188,189]. RKIP was found to be significantly reduced in 42.2% of patients with renal carcinoma (RCC), while this reduction was positively correlated with the presence of distant metastasis occurring [188]. Accordingly, Hagan et al. detected sufficient RKIP levels in primary breast tumors; however, RKIP expression was completely lost in lymph node metastases, thus supporting the notion that RKIP may serve as a metastasis suppressor gene [186]. From a mechanistic point of view, the same study revealed limited correlation between RKIP expression and RKIP’s impact on ERK pathway, suggesting that RKIP may utilize other signaling pathways, such as the NF-kB pathway, along with ERK signaling to regulate breast cancer metastasis [186]. Hagan et al. (2005) noted the potential role of NF-kB because the downregulation of RKIP in MCF7 breast carcinoma cell lines resulted in enhanced phosphorylation and degradation of IkB, which ultimately activates the NF-kB pathway [186]. Thus, it is possible that in breast carcinoma cells, RKIP targets the activation of the EMT through regulation of the NF-kB pathway. 

In this context, Chatterjee et al. found decreased RKIP expression levels in primary prostate tumors as compared to normal counterparts [185]. The findings differ slightly from those of Hagan et al. [186] as RKIP expression decreased in the primary prostate tumors, which did not occur in primary breast cancer tumors, thus suggesting that RKIP reduction may follow a cancer-type-specific manner. The same study also explored whether the metastatic potential of tumor cells could be decreased, or prevented entirely, via chemotherapy treatments that induce RKIP expression. Given the different RKIP profiles in primary and metastatic tumors, RKIP may have varied successes in decreasing the metastatic potential due to the various signaling pathways involved between itself and the EMT process. 

Moreover, decreased RKIP mRNA levels were also detected in transitional cell carcinoma (TCC) of urinary bladder compared to normal bladder tissues [187]. In the study, Zaravinos et al. identified the RAS/MEK/ERK pathway to be involved in the relationship between RKIP mRNA levels in TCC and normal bladder tissues [187]. Hence, in the case of TCC, RKIP may be able to regulate EMT events through the RAS/MEK/ERK pathway. The downregulated RKIP expression in primary TCC tumors aligns with the findings of Chatterjee et al., reinforcing the potential of RKIP-mediated treatment in reducing the metastatic potential of tumor cells. Similarly, Martinho et al. identified an inverse association between RKIP expression and cancer metastasis in endometrial cancer. The research further observed a significant reduction in RKIP expression as endometrial tumors evolved towards a malignant phenotype [190]. A different study by the same group also observed the same trend regarding the absence of RKIP protein expression in glioma tumors. In vitro studies on RKIP silencing in glioma cell lines further validated the association between RKIP inhibition and increased migration of glioma cells [191]. 

### 8.1. Correlation between RKIP Expression and Patients’ Prognosis 

The downregulated expression of RKIP in RCC tissues was significantly correlated with poor overall and disease-free survival in RCC patients [188]. Concomitantly, Chatterjee et al. observed a positive correlation between cytoplasmic RKIP levels in tissues of gastric adenocarcinoma and patient survival probability, thus suggesting the possible prognostic significance of RKIP levels within this cancer type [192,193]. 

Contrarily, the study by Martinho et al. failed to establish any correlation between RKIP expression and patients’ survival rates in endometrial cancer tissues [190]. Interestingly, Martinho et al. found that the absence of RKIP was strongly associated with a poor prognosis in patients with malignant gliomas. In particular, the lack of RKIP expression was related to a highly malignant phenotype and poor survival outcomes of glioma patients [191]. These disparities suggest that the prognostic significance of RKIP expression might depend on the type of cancer being evaluated; however, further research is necessary to validate this notion. 

The relationship between RKIP levels and patient prognosis was further evaluated in a study focused on gastrointestinal stromal tumors (GISTs) [194]. The study found that RKIP expression in GISTs was significantly related to tumor size, the National Institutes of Health (NIH) risk grade, and mucosal invasion, but not significantly associated with age, gender, or tumor location [194]. Additionally, the study observed significantly higher survival rates among patients with higher RKIP expression levels compared to those with low RKIP expression. It also identified NIH risk grade as a significant predictor of GIST prognosis [194]. Since RKIP was significantly related to NIH risk grade, it suggests that RKIP is an excellent predictor of higher NIH risk grades and might indirectly be an effective consideration for predicting the prognosis of GISTs. In another study, Lamiman et al. (2014) reported the clinical evaluation of RKIP as a prognostic value for overall survival, DFS, and presence of metastasis. However, RKIP was only associated with tumor-grade and stage in 50% of patients [195]. 

### 8.2. Potential Use of RKIP as a Diagnostic Biomarker 

Although several studies suggest that RKIP can potentially serve as a diagnostic biomarker, RKIP levels might be considered independently of other major clinical markers, such as tumor differentiation grade, size, and estrogen receptor status [186]. This independence is significant because RKIP’s positive relationship with survival suggests that RKIP levels could be directly measured to potentially predict metastasis. Likewise, the study by Martinho et al. identified the elimination of RKIP expression as an independent prognostic marker in glioma cells [191]. The absence of RKIP as an independent prognostic marker highlights its potential application in treatment plans, as RKIP levels can be solely analyzed as an indicator of cancer metastasis without concerns of additional biomarkers interfering with the analysis. 

The findings by Wang et al. [194] further highlight RKIP’s potential as a diagnostic biomarker for GISTs. Since higher RKIP expression was associated with increased survival probability and can serve as a reliable predictor of NIH risk grades, clinical interventions could utilize RKIP levels as a biomarker to predict the overall survival rate of GIST patients. 

## 9. Bioinformatic Analysis 

**Figure 11:**
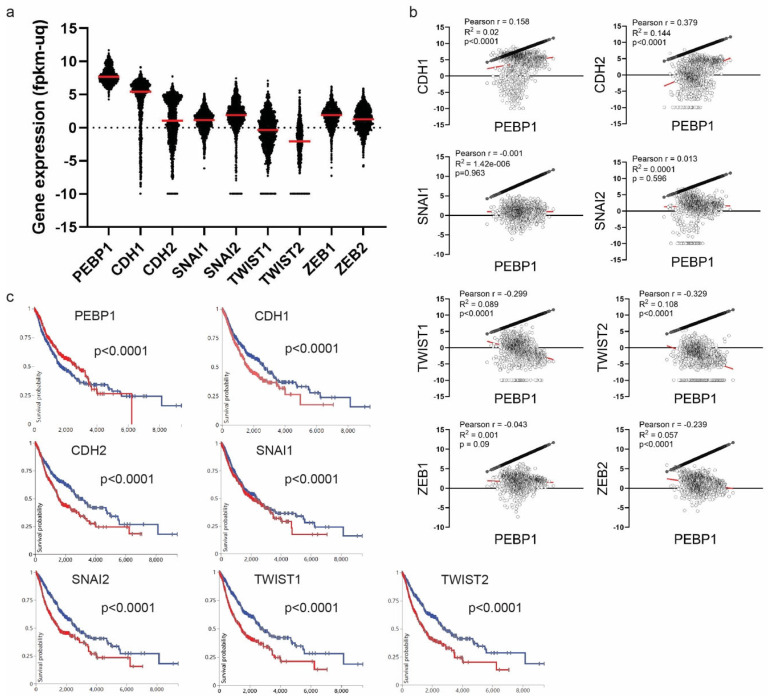
Correlation of mRNA expression between RKIP and CDH1/2, SNAI1/2, TWIST1/2, and ZEB1/2. (**a**) mRNA expression (FPKM-UQ) of RKIP (PEBP1), cadherin 1/2 (CDH1/2), SNAI1/2, TWIST1/2 and ZEB1/2. Data were extracted from 1284 samples from the PCAWG (specimen centric) study. (**b**) Pearson’s correlations between the mRNA expression (FPKM-UQ) of RKIP (PEBP1) and CDH1/2 or the transcription factors SNAI1/2, TWIST1/2 and ZEB1/2. Red line, linear regression. (**c**) Overall survival probability of patients expressing high (red color) and low (blue color) levels of PEBP1 (RKIP), CDH1/2, SNAIL1/2, or TWIST1/2 in pan-cancer. High PEBP1 expression is associated with good prognosis in pan-cancer, while high levels of CDH1/2, SNAI1/2, TWIST1/2 and ZEB1/2 are associated with a poor patient prognosis in pan-cancer (*p* < 0.0001, Log-rank test). Red curves: high expression; blue curves: low expression.

Bioinformatics analysis of 1284 samples extracted from the PCAWG (specimen centric) study showed that PEBP1 (RKIP) mRNA levels were higher and correlated positively with those of CDH1 and CDH2 but negatively with those of SNAI1, SNAI2, TWIST1, TWIST2, ZEB1 and ZEB2 in pan-cancer (Figure 11a,b). In addition, high PEBP1 expression was associated with good prognosis, in pan-cancer, while high levels of the other markers were associated with a poor prognosis (Figure 11c).

## 10. Epithelial and Mesenchymal Biomarkers Targeted Therapeutic Approaches for RKIP Induction

### 10.1. Agonists of RKIP 

Although there are no agents that directly target the upregulation of RKIP, previous studies have found a few agents that indirectly target RKIP. A study by Baritaki et al. highlighted the proteasome inhibitor NPI-0052 (marizomib) as a molecule agonist of RKIP [196]. The study treated human prostate cancer cell lines with NPI-0052 specific-treatment and found induced expression of RKIP, ultimately reversing the chemo-immune resistance when RKIP was inhibited [196]. It is also significant to note that the treatment with NPI-0052 resulted in the inhibition of NF-kB and SNAIL1 [196]. The study’s findings align with the previous findings because inhibition of the NF-kB signaling pathway would partially decrease SNAIL1 expression and SNAIL1 acts as an inhibitor of RKIP, leading to the increase in RKIP expression. 

Another indirect molecule agonist of RKIP is DETANONOate, a common nitric oxide (NO) donor [155]. A study by Bonavida and Baritaki explored the role of DETANONOate as a NO donor in the NF-κB/SΝAΙL/YY1/RKIP circuitry, ultimately finding NO as an indirect agonist of RKIP [175]. The study found that treatment of metastatic cancer cells with DETANONOate induced RKIP expression while inhibiting both NF-κB and SNAIL [175]. It appears that NO shares a similar indirect mechanism with fellow agonist NPI-0052 as the inhibition of NF-κB would result in decreased expression of SΝAΙL and increased expression of RKIP, both gene products affected by the NF-κB signaling pathway. 

A natural agonist of RKIP is epigallocatechin gallate (EGCG), which was found to increase RKIP expression in pancreatic adenocarcinoma cells by inhibiting both NF-κB and SNAIL [197]. A study by Hu et al. found dihydroartemisinin, a derivative of artemisinin and a known antimalarial treatment option, to act as an RKIP agonist in cervical cancers [198]. Two other agonists include American ginseng, which was shown to enhance RKIP expression in breast cancer cells, and the didymin flavonoid in neuroblastoma, which induced RKIP levels while inducing apoptosis of cancer cells [199,200].

Yet, another indirect RKIP agonist is anti-CD20 LFB-R603 [201] and Rituximab, an anti-CD20 monoclonal antibody that was found to increase RKIP expression in B-cell non-Hodgkin lymphoma (NHL) cells [155,202].

### 10.2. Photodynamic and Gene Therapies to Enhance RKIP Expression 

One form of therapy that enhances RKIP expression is the photodynamic therapy (PDT). Gupta et al. found that PDT activated the production of NO from inflammatory cells, while Rapozzi et al. determined a positive relationship between PDT and NO, a known RKIP agonist [155,203,204]. The same study by Rapozzi et al. expanded the relationship by noting that PDT activity along with DETANONOate treatment resulted in the enhanced expression of RKIP [204]. DETANONOate has already been described as an indirect agonist of RKIP and, based on the findings by Rapozzi et al., it is clear that a form of therapy includes the combination of DETANONOate with PDT [204]. The combination therapy essentially increases NO levels, which then enhances RKIP expression while inhibiting NF-kB and SNAIL. The inhibition of NF-kB is intriguing because NF-kB plays a significant signaling role in all the major EMT TFs addressed in this paper, including SNAIL1, ZEB1, ZEB2, TWIST1, and TWIST2. 

Unfortunately, there are not many gene therapies that currently target the enhancement in RKIP expression. Considering the importance of RKIP expression in regulating cancer metastasis via inhibition of EMT TFs, further research on gene therapies should be conducted that specifically target the increase in RKIP expression. 

### 10.3. Immunotherapeutic Strategies Targeting RKIP 

As noted previously, Rituximab is a monoclonal antibody that has been clinically applied, both individually and with chemotherapy, to treat non-Hodgkin’s lymphoma [155,202]. Rituximab acts as an immunotherapy strategy for targeting RKIP because the antibody specifically upregulates RKIP expression, which then proceeds to inhibit both ERK1/2 and NF-κB signaling pathways [202].

There are currently no direct immunotherapy strategies that directly target RKIP. Considering the many inverse relationships that RKIP shares with the major EMT TFs, potential immunotherapy strategies should focus on known regulators of EMT TFs. For example, the various microRNAs that target major EMT TFs could be investigated to create strategies that increase RKIP expression while inhibiting EMT TFs’ expression.

## 11. Challenges and Limitations

### 11.1. Heterogeneity of RKIP Expression in Cancer 

One limitation in relation to any potential RKIP-mediated treatment is the heterogeneity of RKIP expression levels in various carcinomas. The heterogeneity of RKIP expression was explored by Zaravinos and colleagues in a study about RKIP mRNA expression among 37 cancer types based on data from The Cancer Genome Atlas (TCGA) platform [205,206]. The study’s analysis found RKIP to be highest in adrenocortical carcinoma (ACC), liver hepatocellular carcinoma (LIHC), and thyroid carcinoma (THCA), while RKIP was lowest in acute myeloid leukemia (LAML), esophageal carcinoma (ESCA), and stomach and esophageal carcinomas (STES) [205]. The varying differences in RKIP mRNA expressions signify a potential limitation of RKIP-mediated treatment plans as the effectiveness of treatment might vary based on the cancer type and its respective RKIP expression levels. 

Considering that low RKIP levels are associated with worse patients’ prognosis, a hypothetical scenario includes RKIP-targeted therapies being more effective in cancer types such as ACC and LIHC because of the existing higher levels of RKIP as compared to others. Likewise, another hypothetical scenario includes RKIP-mediated therapies being less effective in cancer types with lower RKIP mRNA, such as ESCA and STES, because of the significantly low RKIP levels that are associated with cancer metastasis events. These two hypotheses, though untested, highlight possible limitations of RKIP as a result of its heterogeneity in cancer types and should be taken into consideration in future studies concerning RKIP-mediated treatments. 

### 11.2. Resistance Mechanisms to RKIP-Targeted Therapies 

Studies by Raquel-Cunha et al. and Lai et al. confirmed that decreased RKIP expression is the result of transcriptional and post-transcriptional mechanisms instead of altered or deletion mutations [206,207]. Confirmation of transcriptional and post-transcriptional regulations offers potential in RKIP-targeted therapies, but it also brings to light the possibility of resistance mechanisms to such therapies. The transcription factors of SNAI1, BACH1, and potentially YY1 share a negative regulatory mechanism with RKIP. The study by Papale et al. reported the role of SNAI1 and BACH1 as negative regulators of RKIP, specifically describing the two transcription factors as selectively targeting RKIP in cancer cells [208]. 

On a similar note, this study reported miRNA-27a, miRNA-543, miRNA-23a, and miRNA-224, in certain carcinomas, as post-transcriptional regulators of RKIP. Thus, potential RKIP-targeted therapies may face resistance mechanisms by cancer cells via the upregulation of these transcriptional and post-transcriptional regulators, ultimately leading to decreased effectiveness of such therapies. Another concern regarding resistance mechanisms is based on RKIP expression being lost in secondary tumors after metastasis has occurred. The loss of RKIP expression in secondary, not primary, tumors is a concern because RKIP-targeted therapies would target secondary tumors, but if resistance to such therapies was conferred to secondary tumors, the treatment would be ineffective in inhibiting further invasion and mobility of metastatic tumors. 

## 12. Future Directions

### 12.1. Emerging Research on RKIP and EMT

Although several studies have speculated RKIP’s importance in many cancer types, especially as RKIP is associated with worse prognosis outcomes, few to none have conducted in vivo RKIP-inducing experiments to test its effectiveness against cancer types. The emerging research by Wang et al. is one experiment that has tested RKIP’s potential by transfecting a plasmid meant to induce overexpression of the RKIP gene within cells from the A549 cell line [209]. The integrated plasmid tested how overexpression of RKIP would impact the metastasis of non-small cell lung cancer (NSCLC) by inhibiting EMT [209]. The study’s data revealed that over-expression of RKIP inhibited both the invasion and proliferation of NSCLC cells from the A549 cell line [209]. 

Furthermore, Wang et al. confirmed that RKIP decreased the metastasis of NSCLC via EMT by observing increased levels of E-cadherin, an epithelial biomarker, and decreased levels of vimentin, a biomarker of mesenchymal cells [209]. This emerging study shows the significant promise that RKIP-targeting treatments may have against metastasis events among different cancer types. Considering the heterogeneity of RKIP expression in cancer types, future studies should deploy similar procedures, a plasmid that triggers overexpression of the RKIP gene, in distinct cancer types to evaluate the effects on tumor invasion and metastasis. Moreover, future clinical trials should evaluate how RKIP inhibits EMT, specifically looking at changes in levels of SNAI1, SNAIL2 (SLUG), ZEB1, ZEB2, TWIST1, and TWIST2 transcription factors. 

Another significant research study by Lai et al. utilized gene expression data of human breast tissues to identify the mechanisms behind the loss of RKIP in breast cancer cells [207]. Comparing RKIP expression in breast cancer samples from both tumors that had metastasized and primary tumors, the study found a non-significant amount of altered or mutated copy number alterations in the RKIP gene, suggesting that the loss of RKIP was the result of transcriptional and post-transcriptional mechanisms [207]. This emerging study supports the idea of further studying the transcriptional factors of SNAIL1, ZEB1, ZEB2, TWIST1, and TWIST2 because the loss of RKIP, at least in breast cancer cells, is mediated through transcriptional mechanisms that could potentially be initiated via these transcription factors. Therefore, future studies and medicines could target these strategic transcription factors to inhibit the role of EMT and upregulate expression of RKIP in secondary tumors. In fact, Lai et al. identified Epirubicin as a drug that significantly induced RKIP levels when treating MCF-7 and MDA-MB-231 cells, two types of human breast cancer cell line, within a 24 hour period [207,210]. Although there are currently not many RKIP-targeting treatments, Epirubicin is a positive example of the promises that such therapies and medicines may have against several cancer types. 

Kyakulaga et al. identified Withaferin A (WFA), a naturally occurring 28-carbon compound, as an inhibitor of EMT in non-small cell lung cancer cells [211,212,213]. Based on their findings, it appears that WFA-mediated inhibition of EMT occurs to an extent because of WFA regulation of SNAIL1 expression via SMAD and NF-kB signaling. The potential of WFA as an EMT inhibitor has been shown in other studies, including in breast [211,214], ovarian [215], lung [216], and melanoma cancer cells [217]. Besides WFA, Anwar et al. highlighted several other natural products that could potentially target EMT and serve as anticancer therapeutics [218,219,220]. Resveratrol, a polyphenol in grapes and berries, has been shown to block EMT in malignancies [218,221] and inhibited EMT in glioblastoma cells [222]. The study by Zhou et al. [223] indicated that Paeoniflorin downregulated EMT activity by regulating HIF-1 expression, which is vital in hypoxia-induced EMT. Dihydroartemisinin, originally an anti-malarial agent used to treat drug-resistant malaria strains, has anti-cancer characteristics and can initiate apoptosis in leukemic cells [218,224]. In addition, it inhibits gastric cancer invasion and migration by inhibiting SNAIL1, a key EMT transcription factor. Dihydroartemisinin is one example of how drugs designed to treat other diseases can be repurposed to treat cancers by targeting EMT.

### 12.2. RKIP Potential for Personalized Medicine Approaches 

The concept of personalized precision medicine is based on identifying markers that can be utilized in the clinical setting for early diagnosis, prognosis, and targeted therapy [225,226]. RKIP has been described as an inhibitor of EMT and cancer metastasis, is found at low levels in many cancer types and associated with worse prognosis, and serves as a regulator of several significant transcription factors, all of which suggest that RKIP has tremendous potential for personalized precision medicine. Vicarelli et al. compared RKIP levels between tumor and normal matching samples to evaluate the diagnostic potential of RKIP, ultimately finding that RKIP was significantly downregulated in the tumor samples of lung squamous cell carcinoma (LUSC) and lung adenocarcinoma (LUAD) [225]. The difference in RKIP levels between healthy and cancerous samples supports RKIP as a valuable target for early diagnosis. 

In regard to RKIP levels and prognosis, several cancer studies established an association between low RKIP levels and poor patient prognosis. Although few RKIP-targeted therapies currently exist, the clinical trial by Wang et al. [209] showcased the viability of RKIP-targeted therapies as the transfected plasmid that triggered overexpression of RKIP resulted in the inhibition of invasion and cancer metastasis of NSCLC cells. Considering the requirements that define personalized precision medicine, RKIP has tremendous potential for personalized medicine as a result of how universally applicable the biomarker is and the fact that low RKIP expression is associated with low prognosis outcomes. 

### 12.3. Integration of RKIP Targeting Current Cancer Treatment 

One potential avenue for the integration of RKIP-targeting therapies is first utilizing RKIP as a diagnostic biomarker for cancer types. Medical professionals currently use the NIH 2008 risk classification system to perform prognostic evaluations of gastrointestinal stromal tumors (GIST) patients and determine whether targeted therapies are necessary [227,228]. The use of this classification system is complex, sometimes being difficult to utilize and interpret. Acknowledging the flaws in the current risk classification system, the integration of RKIP-targeting offers significant promise because it is clearly a major biomarker that is downregulated in metastasis events for many cancer types. Thus, integrating RKIP-targeting with current treatment plans has the potential to improve diagnostic evaluations for cancer patients and improve the efficiency of treating cases with worse prognosis. 

Beyond the potential of RKIP as a diagnostic biomarker, RKIP-targeting treatments can be integrated with current cancer treatments to improve patient prognosis. It should be noted that there are currently no in vivo studies that have integrated RKIP-targeting treatments with existing cancer treatments. However, some studies have tested RKIP-targeting medicines to certain cancer types in vitro and found positive results. One example is the study by Lee et al., which treated A549 cells with adriamycin and 9NC, two chemotherapeutic agents, and resulted in induced RKIP expression [229]. Another RKIP-targeting treatment is gemcitabine, a chemotherapeutic drug that induces RKIP expression in the A549, CALU-1, CALU-9, H23, and HCC 827 cell lines [230]. Both studies found increased RKIP expression, highlighting the ability of RKIP-targeting treatments to be integrated in existing treatment options. Importantly, further studies are needed to determine other drugs that can target and upregulate RKIP expression in other cancer types, and can describe how to best integrate into existing cancer treatment schemes. 

## 13. General Discussion

EMT enables epithelial cells to lose their epithelial traits in favor of mesenchymal characteristics during distinct biological periods. Moreover, the EMT phenotype is known to be resistant to chemotherapy and immunotherapy. The first two EMT types (type 1 and type 2) are active during development and periods of wound healing, while type 3 is associated with uncontrolled growth of cancer cells that utilize the process to gain mobility and invasiveness. Hence, targeting EMT is a therapeutic approach that could potentially reverse resistance and inhibit tumor growth and metastasis. 

EMT is regulated by several major gene products, including SNAIL1, SLUG, ZEB1, ZEB2, TWIST1 and TWIST2. This report addressed the transcriptional and post-transcriptional regulatory mechanisms of each of these gene products. Furthermore, the transcriptional and post-transcriptional regulation of RKIP, a gene product that inhibits EMT, was described. RKIP and many of the previously stated EMT transcription factors are involved in the same signaling pathways, enabling cross-talks in this paper about shared systems and inverse relationships. There are still very few RKIP-targeting treatments as well as a lack of in vivo studies that analyze the inhibitory role that RKIP plays in cancer metastasis events. Taking into consideration previous findings regarding low RKIP and poor patients’ prognosis, future studies should investigate new methods of specifically targeting and upregulating RKIP to inhibit cancer proliferation and invasion.

We have summarized the reported literature on the relationship between RKIP and EMT and are aware that these were correlations only and did not address the underlying potential mechanism of this correlation. In the current review, we have discussed that RKIP is involved in the regulation of key TFs that regulate EMT with additional data from bioinformatic analyses. Past studies have set a foundation for the hypothesized relationship between RKIP and EMT. Baritaki et al. (2010) established an early relationship between RKIP and EMT via inhibition of SNAIL1 by DETANONOate. The mechanism was evaluated by overexpressing RKIP and inhibition of EMT [231]. A study by Hill et al. (2014) determined that RKIP knockdown resulted in increased Vimentin, a key EMT biomarker, expression in A498 cells, while the upregulation of RKIP led to decreased Vimentin expression [232]. The study suggested that RKIP limits the invasiveness of clear cell renal cell carcinomas to a certain extent by inhibiting EMT [232]. Shyam et al. (2023) also proposed a relationship between RKIP and EMT through a regulatory network involving BACH1 and RKIP. After finding a negative correlation between RKIP and BACH1 in 31 different cancer types, they established the concept of a back-and-forth battle played out between an anti-metastatic ‘group’ led by RKIP and a pro-metastatic, such as EMT-inducing, led by BACH1 [233]. Noh et al. (2016) established an RKIP/EMT relationship by identifying an inverse relationship between RKIP and NOTCH1 intracellular domain (NICD), which is known to stimulate EMT [158]. Wang et al. (2019) investigated whether RKIP could inhibit the invasion of lung cancer in vitro via the transfection of an RKIP plasmid. The findings suggested that high RKIP expression inhibited EMT, thus producing the change in expression levels of both gene products [209]. While these studies did not examine the underlying potential mechanism of the inverse correlation between RKIP and EMT, we have discussed that RKIP is involved in the regulation of the specific metastatic TFs that regulate EMT and metastases. We suggest, however, that further investigations are warranted to establish the direct role of RKIP in EMT in various cancers as each individual cancer may behave differently.

## Figures and Tables

**Table 1:** Biomarkers’ expression in epithelial and mesenchymal cells.

Biomarkers	Expression in Epithelial Cells	Expression in Mesenchymal Cells	Source(s)
E-cadherin	Yes	No	[37]
Claudins	Yes	No	[38]
Occludins	Yes	No	[38]
Zona-Occludens 1 (ZO-1)	Yes	No	[38]
Mucin-1 (MUC1)	Yes	No	[36,39]
N-cadherin (CDH2)	No	Yes	[36]
Vimentin	No	Yes	[38]
Fibronectin (FBN)	No	Yes	[40]
Fibroblast-specific protein 1 (FSP1)	No	Yes	[41]

## Abbreviations

ACC	Adrenocortical Carcinoma
Ala	Alanine
AML	Acute Myelogenous Leukemia
AR	Androgen Receptor
ARE	Androgen Responsive Element
AVB1	Alpha-V-Beta-1 Integrin
AVB6	Alpha-V-Beta-6 Integrin
BACH1	BTB Domain and CNC Homolog 1
β-Catenin	Beta-Catenin
bHLH	Basic Helix–Loop–Helix
BMP	Morphogenetic Protein
BRG1	Brahma-related Gene 1
CDH1	*E-cadherin* Gene
CDH2	*N-cadherin* Gene
CFPAC-1	Cystic Fibrosis Pancreatic Adenocarcinoma Cell line
ChIP	Chromatin Immunoprecipitation
CTBP	C-Terminal-Binding Protein
DDR2	Discoidin Domain Receptor Tyrosine Kinase 2
DHT	Dihydrotestosterone
E-box	Enhancer-box
ECM	Extracellular Matrix
EGCG	Epigallocatechin Gallate
EGF	Epidermal Growth Factor
EMT	Epithelial-to-Mesenchymal Transition
ERK	Extracellular Signal-Regulated Kinases
ESCA	Esophageal Carcinoma
EZH2	Enhancer of Zeste Homolog 2
FBN	Fibronectin
FBXL14	F-box and Leucine-rich Repeat Protein 4
FGF	Fibroblast Growth Factor
FSP1	Fibroblast-Specific Protein 1
GF	Growth Factor
GIST	Gastrointestinal Stromal Tumors
GSK-3β	Glycogen Synthase Kinase-3-Beta
HDACi	Histone Deacetylase Inhibitor
HEK-293T	Human Embryonic Kidney 293T cells
HGF	Hepatocyte Growth Factor
HIF-1	Hypoxia-Inducible Factor
HIF-1a	Hypoxia-Inducible Factor 1-alpha
HIF-1b	Hypoxia-Inducible Factor 1-beta
HMGA 2	High-Mobility Group A2
IGF	Insulin Growth Factor
IkB	Inhibitors of κB
LAML	Acute Myeloid Leukemia
LATS2	Large Tumor Suppressor Kinase 2
LIHC	Liver Hepatocellular Carcinoma
LncRNA	Long Non-Coding RNA
LOXL2	Lysyl Oxidase Like 2
LRP	Lipoprotein Receptor Protein
LSD1	Lysine-Specific Demethylase 1
LUAD	Lung Adenocarcinoma
LUSC	Lung Squamous Cell Carcinoma
MAPK	Mitogen-Activated Protein Kinase
MDCK	Madin-Darby Canine Kidney cells
MEK1	Mitogen-Activated Protein Kinase 1
MEKK1	Mitogen-Activated Protein Kinase Kinase 1
MET	Mesenchymal–Epithelial Transition
miRNA	MicroRNA
MMSET	Multiple Myeloma SET Domain
MRTF	Myocardin-Related Transcription Factor
MUC1	Mucin-1
NES	Nuclear Export Sequence
NF-κB	Nuclear Factor Kappa B
NHL	Non-Hodgkin Lymphoma
NIC	Notch Intracellular Domain
NIH	National Institutes of Health
NIK	NF-kB Inducing Kinase
NO	Nitric Oxide
NPI-0052	Marizomib
NSCLC	Non-Small-Cell Lung Cancer
O-GlcNAc	Hyperglycemia-Regulated O-linked B-N-acetylglucosamine
PAK1	p21-Activated Kinase
PCAF	P300/CBP-Associated Factor
PDGF	Platelet-Derived Growth Factor
PDT	Photodynamic Therapy
PI3K	Phosphoinositide-3-Kinase
PKA	Protein Kinase A
PRC	Polycomb Repressor Complex
RCC	Renal Cell Carcinoma
TGF-B	Transforming Growth Factor-Beta
TGF-BR	Transforming Growth Factor-Beta Receptor
RKIP	Raf Kinase Inhibitor Protein
RTK	Receptor Tyrosine Kinase
S68	Serine 68
S68A	Serine 68 Mutation
SCP	Small C-Terminal Domain Phosphatase
SMAD	Small Mother Against Decapentaplegic
SRD	Serine-Rich Domain
STES	Stomach and Esophageal Carcinomas
TAK1	Transforming Growth Factor Beta-Activated Kinase 1
TCC	Transitional Cell Carcinoma
TCGA	The Cancer Genome Atlas
TF	Transcription Factor
THCA	Thyroid Carcinoma
TNBC	Triple-Negative Breast Cancer
TNF	Tumor Necrosis Factor
TSA	Trichostatin A
UTR	Untranslated Region
Wnt	Wingless-Related Integration Site
YY1	Ying-Yang 1
ZEB	Zinc Finger E-box Homeobox
ZO-1	Zona-Occludens 1
